# A general model of hormesis in biological systems and its application to pest management

**DOI:** 10.1098/rsif.2019.0468

**Published:** 2019-08-21

**Authors:** Sanyi Tang, Juhua Liang, Changcheng Xiang, Yanni Xiao, Xia Wang, Jianhong Wu, Guoping Li, Robert A. Cheke

**Affiliations:** 1 School of Mathematics and Information Science, Shaanxi Normal University, Xi'an 710119, People's Republic of China; 2 Department of Mathematics, Hubei University for Nationalities, Enshi 445000, People's Republic of China; 3 Department of Applied Mathematics, Xi'an Jiaotong University, Xi'an 710049, People's Republic of China; 4 Laboratory for Industrial and Applied Mathematics, York University, Toronto, Ontario, Canada M3J1P3; 5 Institute of Plant Protection, Henan Academy of Agricultural Sciences, 116 Huayuan Road, Zhengzhou 450002, People's Republic of China; 6 Natural Resources Institute, University of Greenwich at Medway, Central Avenue, Chatham Maritime, Kent ME4 4TB, UK; 7 Department of Infectious Disease Epidemiology, School of Public Health, Imperial College London, St Mary's Campus, Norfolk Place, London W2 1PG, UK

**Keywords:** ecological paradox, Ricker equation, *Apolygus lucorum*, pest control, complex dynamics, stability

## Abstract

Hormesis, a phenomenon whereby exposure to high levels of stressors is inhibitory but low (mild, sublethal and subtoxic) doses are stimulatory, challenges decision-making in the management of cancer, neurodegenerative diseases, nutrition and ecotoxicology. In the latter, increasing amounts of a pesticide may lead to upsurges rather than declines of pests, ecological paradoxes that are difficult to predict. Using a novel re-formulation of the Ricker population equation, we show how interactions between intervention strengths and dose timings, dose–response functions and intrinsic factors can model such paradoxes and hormesis. A model with three critical parameters revealed hormetic biphasic dose and dose timing responses, either in a J-shape or an inverted U-shape, yielding a homeostatic change or a catastrophic shift and hormetic effects in many parameter regions. Such effects were enhanced by repeated pulses of low-level stimulations within one generation at different dose timings, thereby reducing threshold levels, maximum responses and inhibition. The model provides insights into the complex dynamics of such systems and a methodology for improved experimental design and analysis, with wide-reaching implications for understanding hormetic effects in ecology and in medical and veterinary treatment decision-making. We hypothesized that the dynamics of a discrete generation pest control system can be determined by various three-parameter spaces, some of which reveal the conditions for occurrence of hormesis, and confirmed this by fitting our model to both hormetic data from the literature and to a non-hormetic dataset on pesticidal control of mirid bugs in cotton.

## 1. Introduction

Biological systems exhibit a variety of unexpected, sometimes paradoxical, behaviour. Examples include catastrophic shifts [1] at tipping points in ecosystems [2], phase changes in polyphenic insects [3] and hormesis in toxicology, whereby a cell or organism exhibits a biphasic response when exposed to increasing amounts of a substance or external conditions [4–6]. Ecological paradoxes involving interactions between two or more species include coexistence of competing species [7] and the ‘paradox of the plankton' [8,9]. Other examples arise from repeated applications of pesticides that have unexpected consequences because of Volterra's principle, when an intervention in a predator–prey system that removes predator and prey in proportion to their population sizes increases the prey population [10]. In addition, a food type may become rarer in a diet even when it becomes more abundant [11] and, in single species systems as discussed here, incorrect use of pesticides may not control pests effectively and can also lead to rapid increases in the number of pests, thus inducing bigger outbreaks [12].

Hormetic (paradoxical) effects (HPEs) also pose significant challenges for decision-making in treatments of cancer [13–15] and neurodegenerative diseases [16], and in the management of nutrition [17] and ecotoxicology [18]. Strategic applications of hormetic effects have shown promise in optimizing the management of agro-ecological systems with pesticides [19,20] or harvesting [21–23] in relation to variations in endogenous regulatory mechanisms, intervention timing and dose–response specifics. However, there is a gap separating experimental designs and field observations from simple mathematical models that can be parametrized by experiments and which, when applied, can also exhibit all of the above paradoxical behaviour. Here, we fill this gap by deriving a simple unifying three-parameter model for discrete generation single species populations. It is based on the Ricker equation [24] for continuous population dynamics between observation intervals and is interrupted by an external dose at a particular time between the observations. The model has three critical parameters: the intrinsic growth rate, the dose–response and the dose timing of interventions [25], from which we can see that the time factor plays a fundamental role in designing proper experiments, and in understanding hormetic effects.

We hypothesized that the dose and dose timing can interact in our new model, which would thus be able to reproduce a wide range of hormetic phenomena, and our analytical results did indeed reveal hormetic biphasic dose and dose timing responses either in a J-shape or an inverted U-shape, yielding a homeostatic change or a catastrophic shift. A multi-parameter bifurcation analysis showed that hormetic effects occur in many parameter regions due to different interactions between intrinsic growth dynamics and the strength and timing of external stimulations; hence many more hormetic effects in nature than those currently recognized should be expected. We also use our model to show that hormetic effects can be significantly enhanced under multiple low-level stimulations [26] within one generation, and this enhanced effect may reduce threshold levels, including for the hormetic zone, as well as reducing both the maximum response and inhibition. This shows that multiple and repeated low-level stimulations may not only provide insights into observations of hormetic effects more clearly and quickly, but also reduce associated risks such as those involved in dosage decisions for medical treatments. Data fitting of our model to laboratory and field data demonstrates the effectiveness of our framework for using experimental data to inform intervention strategies for species with discrete generations under multiple impulsive interventions.

## 2. Methods

### 2.1. Model

Consider discrete generations of a single species population, modelled by

2.1 $$N_{n+1}=f(N_{n},r,p,\theta ),$$

where the population size at the (*n* + 1)th generation is determined from its previous generation, subject to internal continuous dynamics with the intrinsic growth rate *r*. The parameter q=1−p  with  p∈(0, 1]  (survival  rate)  is characterized by pesticide or drug dose or effectiveness and describes the pesticide or drug efficacy and is closely related to (but not equivalent to) the applied dosage of pesticides or drugs at *n* + *θ*, θ∈[0, 1]. Thus, for convenience, we call the parameter *q* the dose–response, and the parameter *θ* is accordingly called the dose timing response. Developing a realistic formulation of *f* to describe the combined effects of internal regulation and the dose–response and dose timing response of external simulations on hormesis has long been regarded as a challenging task [18]. Here, we addressed this challenge by using analytical and piecewise methods [27–30] (electronic supplementary material, §1) to derive a formulation of *f* by incorporating impulsive external stimulation into the Ricker model [24]. Our formulation

2.2 $$f(N,\;r,\;p,\;\theta )=pN\exp\left[r\left(1-\frac{N}{K}\left(\theta +(1-\theta )p\exp\left(r\left(1-\frac{N}{K}\right)\theta \right)\right)\right)\right],$$

henceforth referred to as the hormesis Ricker model (HRM), with *K* being the carrying capacity, provides a closed-form description of how the dose–response and dose timing response of external stimulations affect the reproductive capacity. The HRM clearly shows how intraspecific competition affects population growth after the pesticide application at time *θ*, which is markedly different from the models previously proposed [22,23]. From those publications, we can see that a linear combination relation between the growth function before a pesticide application and the growth function after an application has been assumed. This could largely reduce the effects of the intraspecific density regulation on the reproductive capacity once the external stimulation occurs at time *n* + *θ*. Moreover, this simplifying assumption cannot really explain the essence of hormetic and paradoxical effects, which can be verified based on the theoretical analyses shown in the electronic supplementary material.

The two special cases are *θ* = 0 and *θ* = 1, which result in the following two discrete models:

$$N_{n+1}=pN_{n}\exp\left[r\left(1-\frac{\;pN_{n}}{K}\right)\right]$$

and

$$N_{n+1}=pN_{n}\exp\left[r\left(1-\frac{N_{n}}{K}\right)\right].$$

These two formulations correspond to control measures being applied at the beginning and end of a generation, respectively, and, obviously, these two special models could have the same dynamics as those of the classic Ricker model [27–29]. In particular, the above two special discrete models describe the iterative relationship between the number in a population at two successive generations, but do not reflect the population dynamics within the two generations. However, the practical problem is that the external stimulation is often applied within the two generations, rather than at the start or end of the two generations. This is precisely what our new model (2.2) focuses on, i.e. the impact of the external stimulation at specific times within the two generations on paradoxical and hormetic effects. Therefore, the dose–response and dose timing response must act together, which means that in order to reveal the important factors affecting the occurrence of hormetic effects it is necessary to consider the two factors simultaneously. The importance of the dose timing response (i.e. θ∈(0,1)) has been addressed theoretically in the electronic supplementary material.

We assume that model (2.1) has a stable equilibrium $N^{*}(r,q,\theta )$ at which the homeostatic state is normalized to unity when q=0  and  θ=0, that is, $N_{0}^{*}(r,0,0)=1$. The existence and stability of all possible equilibria can be found in the electronic supplementary material. Hormesis related to the dose–response, characterized by low-dose stimulation and high-dose inhibition, is described by the following paradoxical effect: $\partial N^{*}(r,\;q,\;\theta )/\partial q>0$ for small *q*, $\partial N^{*}(r,\;q,\;\theta )/\partial q<0$ for large *q*, and $N^{*}(r,q,\theta )<1$ when *q* is large enough.

### 2.2. Micro-plot field experiment, published hormetic datasets and data fitting

A field experiment was conducted at the Seven Mile Camp experimental base of Yuanyang County of the Henan Academy of Agricultural Sciences in 2012. There were seven experimental fields arranged and numbered sequentially, each with three replicates giving a total of 21 plots. Each plot was 20 m long and 10 m wide with seven replicates such that the total area involved was about 4200 m^2^. The cotton planting array pitch was 1 m between plants with rows spaced 0.28 m apart. Each plot was separated from its neighbour by 2 m, with corn planted in the gaps for quarantine. Different thresholds for spraying were used in each field according to previous control experience.

The gradient of the action threshold was 1, 2, 5, 10, 20, 40 and 60 infested heads/100 plants for invoking spraying in fields 1–7, respectively. The numbers of *Apolygus lucorum* (Hemiptera: Miridae) bugs were recorded and damage estimated every 4–6 days from 28 May to 5 September 2012. When the pest numbers exceeded the given action threshold in each field, the pesticide was applied before the next day (every unit of 2.5% permethrin was dissolved in 1500 units of fogging liquid). In table 1, South 1–7 represent the seven fields. Based on the above action thresholds, the timings of pesticide applications for the seven fields are italics in table 1.

**Table 1.** Data on numbers of cotton plant heads infested per 100 plants in each of the seven experimental fields from 28 May to 5 September 2012. Days when control measures were applied are shown in italic.

date	South 1	South 2	South 3	South 4	South 5	South 6	South 7
28 May 2012	0	0	0	0	0	0	0
3 June 2012	0	0	0	0	0	0	0
7 June 2012	0	0	0	0	0	0	0
12 June 2012	0	0	0	2	0	0	0
18 June 2012	0	0	0	0	0	0	0
22 June 2012	0	0	0	0	0	0	0
27 June 2012	0	1	0	0	0	0	0
2 July 2012	0	0	0	0	1	0	0
7 July 2012	0	0	1	0	0	0	0
12 July 2012	0	0	0	0	0	2	1
17 July 2012	0	*2*	*7*	3	9	2	3
22 July 2012	0	*3*	*5*	5	3	1	3
27 July 2012	*6*	*8*	*5*	1	6	3	10
2 Aug 2012	*6*	*18*	*7*	16	7	*16*	20
7 Aug 2012	*7*	*10*	*12*	*12*	5	13	14
11 Aug 2012	*31*	*12*	*9*	*23*	*51*	*49*	22
16 Aug 2012	*38*	*42*	*18*	*16*	*51*	*80*	*63*
21 Aug 2012	*26*	*28*	*29*	*28*	*27*	*41*	28
26 Aug 2012	*23*	*30*	*19*	*14*	*24*	19	24
31 Aug 2012	*2*	*9*	*8*	5	3	5	5
5 Sep 2012	*3*	*5*	2	3	4	3	3

We emphasize that our experimental field dataset described above is not a hormetic dataset. It is used only for confirming that our model can be fitted successfully to data with single or multiple pulsed external stimulations within each pest generation. In order to confirm that the model can be fitted to hormetic data, we analysed two published datasets. The first concerned effects of applications of the herbicide glyphosate on the growth and yields of chickpea *Cicer arietinum* (fig. 1b in [31])*.* The second involved applications of deltamethrin to an insecticide-resistant strain of the maize weevil *Sitophilus zeamais* (fig. 4 in [32]).

### 2.3. Data fitting

**Figure 1.**
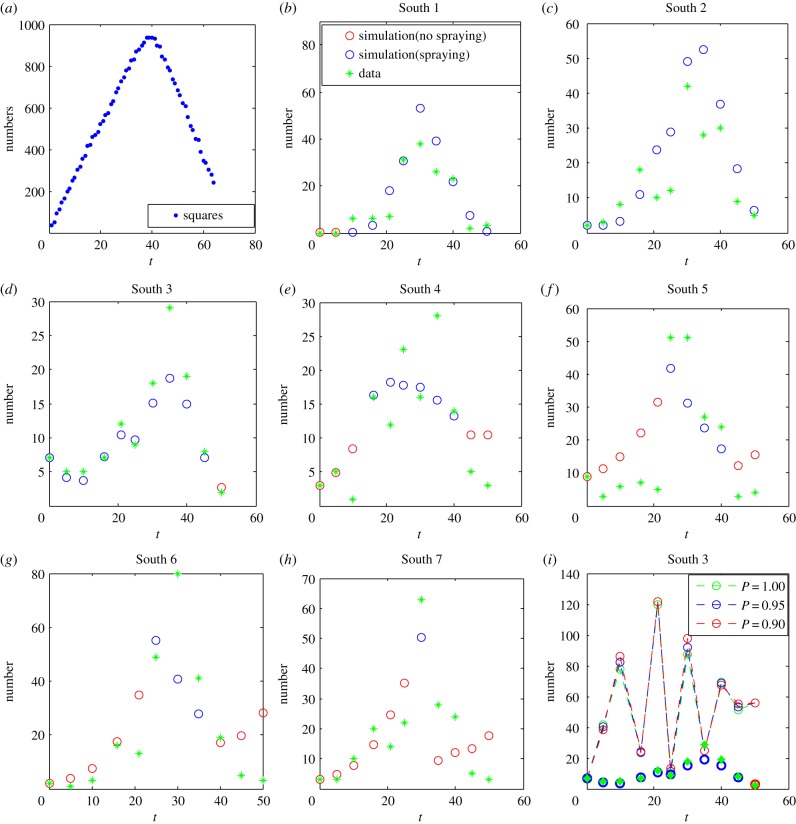
Case study. (*a*) The function *f* for determining the growth rate of the pest population during the plant period. (*b–h*) Results of data fitting for fields South 1–7. (*i*) Effects of dose–response on the pest population number with parameter values $K=125.6677,\bar{r}=0.006,\tau =19.874$, determined by field South 3. Red circles in (*b–h*) represent points when the pesticide was not applied. (Online version in colour.)

First, we assumed that the growth rate of the pests in our field experiment depended on the number of cotton plant squares [33], such that *r*(*t*) = $\bar{r}$*f*(*t* + *τ*), where *τ* represents the phase difference between the numbers of squares and pests, *f* denotes a function for the number of squares with respect to time *t*, and $\bar{r}\;\;$ is the growth rate coefficient. The number of squares with respect to the days after planting is based on data in [33]. Hence, the data for squares can be extracted (as shown in figure 1*a*), and the function can be obtained by using the B spline method. As the earliest date for the pesticide spraying was 17 July, we let the initial time of our model be 17 July. The initial number of pests was the same as for our data. The least-squares method was used to estimate the unknown parameters *p; K;*$\;\;\bar{r}$*; τ,* and the estimations and fitting results for all seven fields are given in table 2 and figure 1*b–h*.

**Table 2.** Parameter estimation results.

	*p*	*K*	$\bar{r}$	*τ*
South 1	0.0933	40.0000	0.0009	30.0000
South 2	0.1441	56.6505	0.0006	22.9071
South 3	0.1000	125.6677	0.0000	19.8743
South 4	0.6617	150.0000	0.00124	30.0000
South 5	0.4983	150.0000	0.0001	14.6434
South 6	0.4342	150.0000	0.000164	87.3513
South 7	0.1352	150.0000	0.0001198	28.0625

**Figure 2.**
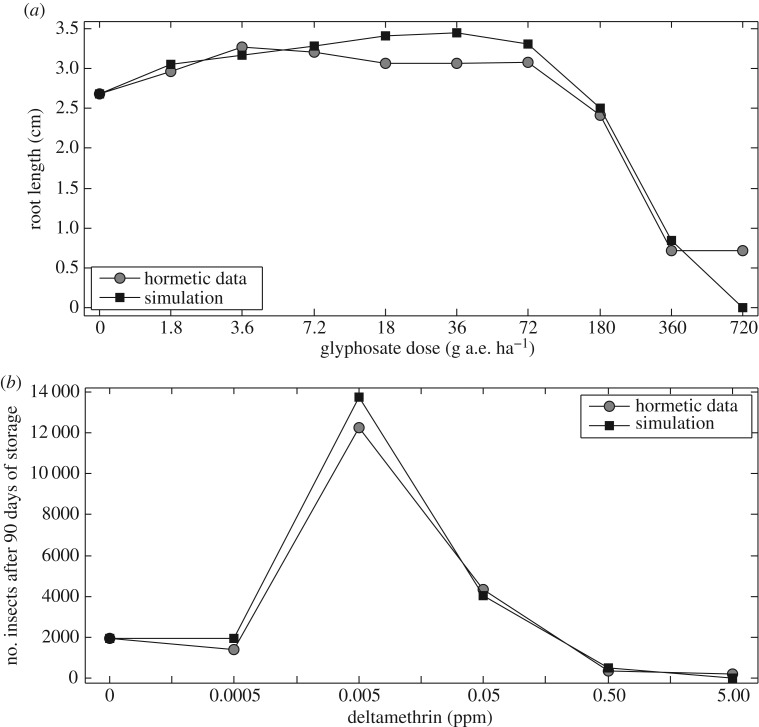
Hormetic data fitting. (*a*) Hormetic effect of glyphosate on growth of chick pea after 21 days spraying, measured by root length. The estimated parameter values are r=4, θ=0.53, ρ=0.09. (*b*) Hormetic effect of deltamethrin on predicted population size of maize weevil. The estimated parameter values are r=4.999, θ=0.1066, ρ=8.08.

Moreover, the published hormetic datasets could be fitted by using similar methods. To do this, we chose the dose–response function $p=e^{-\rho \times Ds}$ to estimate the unknown parameter *ρ* rather than *p*, where *Ds* denotes the glyphosate dose in figure 2*a* and deltamethrin dose in figure 2*b*. Note that the carrying capacity *K* for both hormetic datasets is determined by the first data point (i.e. *Ds* = 0). The dose timing *θ* was determined by the experimental design, for example, the various glyphosate doses were applied four weeks after chick pea emergence, and then the data points were collected after 21 days of glyphosate applications. Therefore, the dose timing *θ* for glyphosate hormesis is about 4/7, which is confirmed by our data fitting and parameter estimation in figure 2*a*. Similarly, the dose timing *θ* for deltamethrin hormesis is confirmed in figure 2*b*, and it is in good agreement with the experimental design.

## 3. Results

### 3.1. Theoretical explorations of the model

In this section, we show how the hypothesis raised in the introduction that the HRM can reproduce a wide range of hormetic phenomena with general applicability is realized.

**Figure 3.**
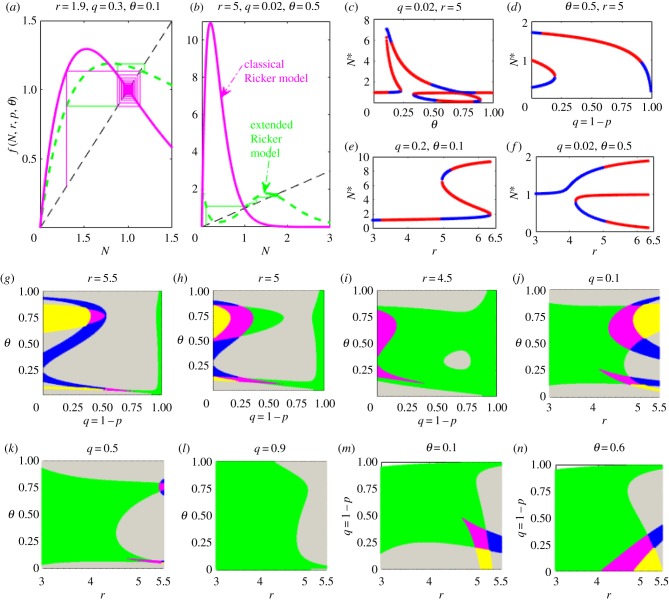
Homeostatic changes and catastrophic shifts. The homeostatic state reaches an altered equilibrium (*a*) and experiences a catastrophic shift to a larger steady state (*b*) when the population is challenged by a hormetic stressor for the HRM. (*c–f*) A sample of single-parameter catastrophic bifurcation diagrams, where a blue/red line represents a stable/unstable equilibrium. The saddle-node or flip bifurcations with respect to dose timing response (*θ*), dose–response (*q*) and growth rate (*r*) show the occurrence of hormetic effects for a wide range of parameter space. (*g–n*) Two-parameter equilibrium bifurcations. White areas denote where no positive equilibrium exists; grey areas indicate where unstable unique positive equilibria exist; green areas indicate single stable equilibria; magenta areas show bistable areas with three equilibria; blue areas represent where three equilibria exist of which only the smallest one is stable, and yellow areas show where three equilibria exist of which only the largest is stable. (Online version in colour.)

Homeostatic changes and catastrophic shifts due to low-level stimulations can be evinced by compensation as a new equilibrium (figure 3*a*) is established or by switching from one stable state to another larger stable state (figure 3*b*), where at a relatively large population size, the reproductive capacity is effectively enhanced when a low-dose external stimulation is applied. The population's intrinsic reproductive capacity is not fully expressed under natural conditions, but a low-dose external perturbation may result in hormetic effects such that the population size is pushed beyond its previous homeostatic state to a new larger equilibrium. The establishment of a higher level equilibrium, as shown in figure 3*b*, is achieved under the dual effects of catastrophic bifurcation and the higher equilibrium stability induced by the low-dose stimulus.

The catastrophic bifurcation diagram figure 3*c* reveals the role of the dose timing response *θ*, in the occurrence of hormetic effects. For low-level stressors, a slight incremental change to *θ* may induce a catastrophic transition to a larger, alternative, stable state. The saddle-node or flip bifurcations with respect to one of the three parameters clearly show the occurrence of hormetic effects for a wide range of parameter values (figure 3*c*). We have also conducted a two-parameter equilibrium bifurcation analysis to examine the synergistic interaction of internal regulation, dose–response and dose timing response. The analysis showed that bi-stability occurs only when *q* is relatively small (the magenta areas in figure 3*g–i*)). Further illustrations based on three-parameter bifurcation analyses are reported in the electronic supplementary material. Note that parallel analyses based on the Beverton–Holt model [34] cannot produce hormetic effects, confirming the importance of the internal regulation mechanism for hormesis to take place (electronic supplementary material, §S1).

When the carrying capacity is normalized to one (*K* = 1), a necessary condition for hormetic effects to occur is f(1, r, q, θ)>1, i.e. the low-level stimulation reduces intraspecific competition to enhance intrinsic reproductive capacity to exceed unity. When this happens, a sufficient condition can be derived (electronic supplementary material, table S1) for the system to shift from a homeostatic state to a higher level equilibrium as *r* < *r_c_*, with the threshold *r_c_* being given by *θ* and *q*, i.e. $r_{c}=1/\theta [\ln(\theta /(1-\theta )p)+2]$. The stability analysis, summarized in electronic supplementary material, table S2, shows how (under the above-threshold condition), low-level stressors induce homeostatic changes/catastrophic shifts. In particular, bi-stability and catastrophic shifts may occur only when the parameter pair (*r*, *p* = 1 − *q*) is within a certain region in the *rp*-parameter space that is separated by the curve r+ln⁡(p)=2 into two parts. Within the region 0<r+ln⁡(p)<2, we can observe different homeostatic modulations: if *r* < 1 (i.e. $N_{0}^{*}(r,\;0,\;0)=1$ is stable), then r+ln⁡(p)<2 holds, and the stable population level can be pushed beyond the normalized value of one to reach a new larger equilibrium $N_{1}^{*}>1$ by the application of a low-level stimulation. If *r* > 2 (i.e. $N_{0}^{*}(r,\;0,\;0)=1$ is unstable), then slightly increasing the stimulation can lead to r+ln⁡(p)<2, inducing the stability of the new larger equilibrium and yielding hormetic effects. Note that, from electronic supplementary material, table S2, we observe that in the region r+ln⁡(p)>2 the system can also be stabilized at the new larger equilibrium $N_{1}^{*}>1$ or $N_{3}^{*}>1$.

**Figure 4.**
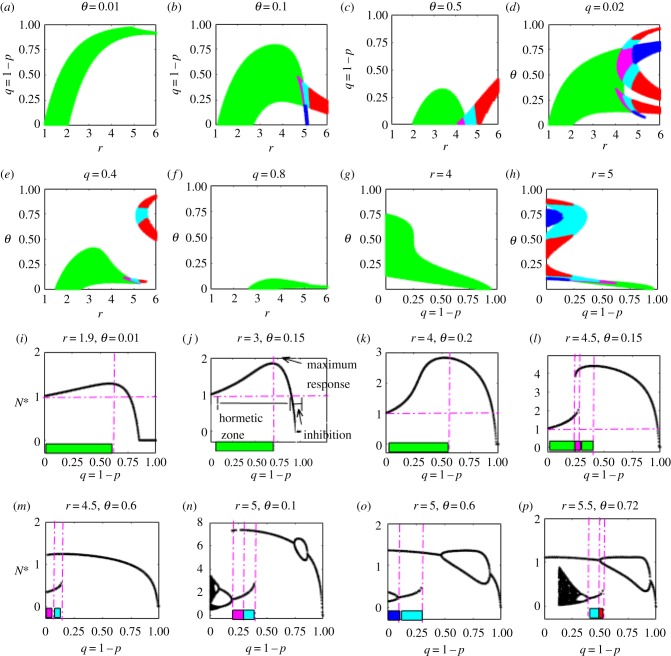
Paradox and hormetic-like biphasic dose–responses. Two-parameter bifurcation spaces for HPEs related to the efficacy of the external stimulation *q* in (*a–h*) for the HRM. Green areas represent where there is a unique stable equilibrium with $\partial N^{*}/\partial q>0$, which indicates that the level of a single stable equilibrium will be increased as *q* increases for a wide range of parameter space. Magenta areas denote bistable regions with $\partial N_{1}^{*}/\partial q>0$ and $\partial N_{3}^{*}/\partial q>0$. Cyan areas indicate bistable regions with $\partial N_{1}^{*}/\partial q>0$ and $\partial N_{3}^{*}/\partial q<0$; red denotes $\partial N_{1}^{*}/\partial q>0$ and $N_{3}^{*}$ is unstable; blue denotes $\partial N_{3}^{*}/\partial q>0$ and $N_{1}^{*}$ is unstable. Homeostatic changes and catastrophic shifts for hormetic-like biphasic dose–responses (i.e. inverted U-shape curves or J-shape curves) are shown in (*i–p*), and the colour bars are consistent with the colour maps in (*a–h*). (Online version in colour.)

The occurrence of paradoxical effects is closely related to the mechanism towards hormesis. The discussion summarized in the electronic supplementary material, tables S3 and S4 for the occurrence of hormetic effects shows that a more refined parameter space is needed, in comparison with those for stability conditions. In particular, electronic supplementary material, table S4 confirms that the dose timing response of external stimulations may have a stronger influence on populations with large intrinsic growth rates. The two-parameter bifurcation diagrams, figure 4*a–h*, show how *r*, *q* and *θ* together influence the stability of equilibria and their variations (i.e. the sign of $\partial N^{*}/\partial q$) when the dose changes (electronic supplementary material, table S3). For example, figure 4*j* shows that increasing the low-level stimulation before reaching the maximum response will result in a stronger paradoxical effect within the hormetic zone. Moreover, the smaller that *θ* and *q* are, the larger are the green areas and, for a relatively small intrinsic growth rate *r*, hormetic effects only occur for extremely small *θ* and *q*. However, when the growth rate *r* is large, the hormetic effects are presented in complex patterns, as illustrated in figure 4*d,h*.

Single-parameter bifurcation analyses, shown in figure 4*i–p*, reveal one of the most common features of hormesis: hormetic biphasic dose–responses (inverted U-shape or J-shape) of low-dose stimulation and high-dose inhibition. There are two types of inverted U-shape curves: one is a continuous inverted U-shape curve (figure 4*i–k*), which reveals the homeostatic changes due to external stimulation as a new and large stable equilibrium is established; the other is a piecewise continuous inverted U-shape curve (figure 4*l–p*), which reveals the importance of catastrophic shifts and the strength of the stability produced by external stimulations in homeostatic changes in generating a hormetic-like biphasic dose–response [2].

**Figure 5.**
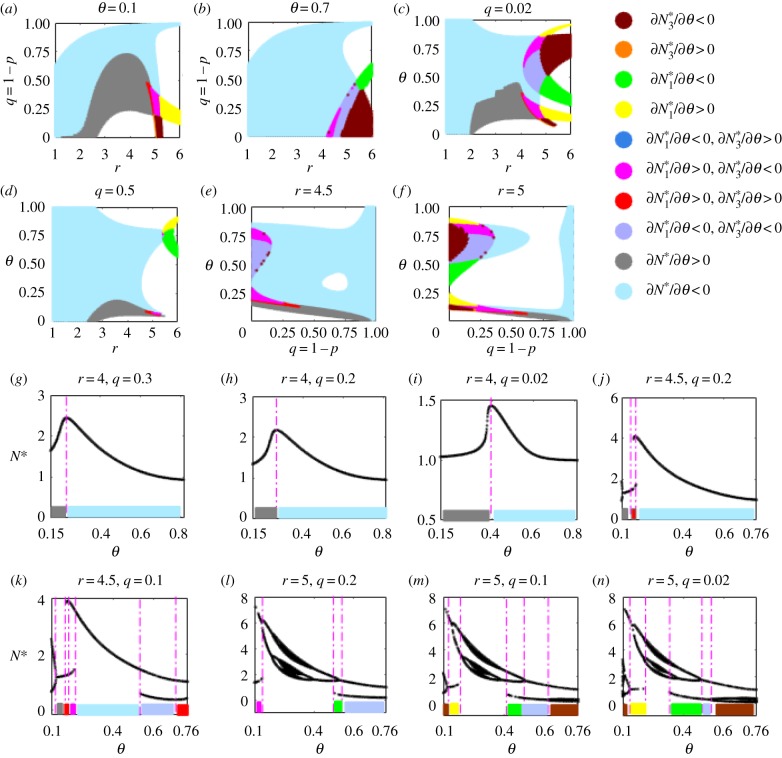
Paradox and hormetic-like biphasic dose timing responses. Two-parameter bifurcation spaces for HPEs related to the timing of the external stimulation *θ* in (*a*–*f*) for the HRM. The signs of the derivative N* with respect to *θ* have been marked in different colours. Homeostatic changes and catastrophic shifts for hormetic-like biphasic dose timing responses (i.e. inverted U-shape curves) are shown in (*g*–*n*), and the colour bars are consistent with the colour maps in (*a*–*f*). The dose timing responses generate inverted *U*-shaped curves similar to those for dose–responses, indicating that the timing could also be an important factor for HPEs. (Online version in colour.)

The dose timing response, *θ*, could also be closely related to the hormetic effects. The two-parameter bifurcation diagrams, figure 5*a–f*, show how *r, q* and *θ* influence the stability of equilibria and their variations when the timing changes. This presents an even more complex pattern in comparison with figure 4*a–h* (electronic supplementary material, table S4). Similarly, a smaller stressor *q*, a larger growth rate *r,* and a different dose timing response can result in very complex patterns, as shown in figure 5*c–f*. Continuous inverted U-shape curves (figure 5*g–i*) and piecewise continuous inverted U-shape curves (figure 5*j–n*) can also be generated by the dose timing response, resulting in hormetic-like biphasic dose timing responses, with earlier dose timing response stimulation, and later dose timing response inhibition.

**Figure 6.**
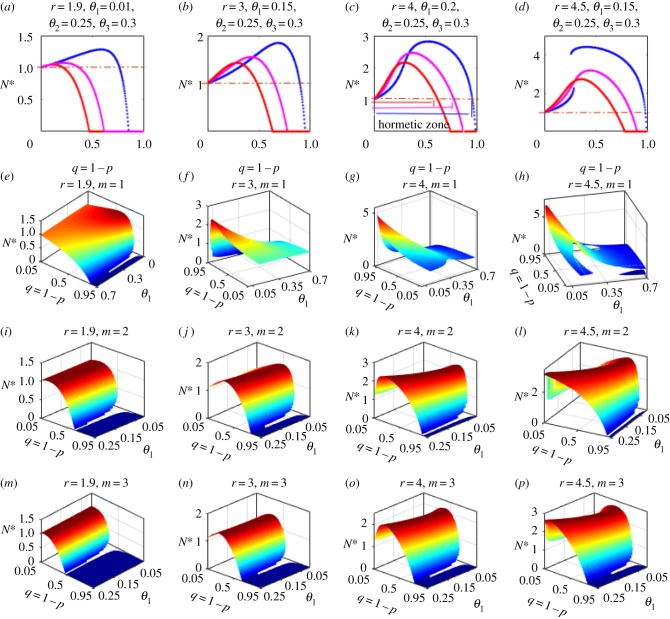
Cumulative effects. Results of applying multiple low-level stimulations at different dose timing responses *θ_i_* with the same dose–response *q* defined in the model (electronic supplementary material, S5.28). (*a*–*d*) Three hormetic dose–response curves with one to three dose–response stimulations at times *θ_i_* (*i* = 1, 2, 3): blue (one dose), magenta (two doses) and red (three doses). Three-dimensional plots for single dose–responses (*e*–*h*), two dose–responses (*i*–*l*) and three dose–responses (*m*–*p*), with different intrinsic growth rates *r*. The baseline parameter values are shown at the top for all subfigures; only the stable equilibria are shown in these subplots. (Online version in colour.)

The hormetic-like biphasic dose–responses and dose timing responses can be significantly modulated and enhanced by multiple applications of low-dose stimulations within each generation at different dose–response times (figure 6). Cumulative effects of multiple low-level stimulations result in a faster increasing of N* for very small values of *q*, and the larger the number of external stimulations, the faster N* increases (figure 6*a–d*). Moreover, the hormetic zones, maximum responses and threshold levels at which the control is effective can be substantially reduced as the number of external stimulations increases. As the dose timing response and dose–response alter, different patterns of equilibrium variation emerge: the spectral ranges for occurrence of hormetic effects significantly change with increasing dose–responses and growth rates (figure 6*e–p*). This reveals a complication of hormetic effects due to the integration of multiple parameters, which poses a serious challenge for evaluating and avoiding the risks produced by hormesis. Nevertheless, there is also a threshold number of dose–responses above which the hormetic effects never occur.

### 3.2. Data fitting to field experiment

We successfully fitted the model using data from the field micro-plot experiment. The intrinsic growth rate of the pest population was determined by proxy from the growth curve of the cotton squares (figure 1*a*). Other unknown parameters of the HRM were estimated, and the best data fits for different experimental plots are shown in figure 1*b–h*. The results clearly show the effects of dose–responses (figure 1*i*): when the low dose is applied, the control is effective at the early stage, but the pest population can increase later, after a few days, to exceed the number when no pesticide is applied. However, a high dose can successfully suppress the pest population to a low level, using the action threshold specified in the field (South 3).

The results confirm that the HRM depicted the field data with multiple pulse control measures well. Note that if we fixed the intrinsic growth rate *r* as a constant and estimated the different survival rates *p* at each observation, we obtained similar results, but this could lead to difficulties for the parameter estimations. The green line in figure 1*i* shows the simulation results without pesticide spraying. The red and blue lines present the simulations when 5% and 10% of the pest are killed after pesticide applications.

The effectiveness of the pesticide in the early stages of low-dose applications and loss of the effectiveness of the pesticide in the late stages confirm that our new model can successfully reveal the occurrence of paradoxical and hormetic effects, predict the potential risks, and provide guidance for designing new experiments on hormetic effects. To confirm these conclusions, we analysed the two hormetic datasets shown in figure 2 to estimate the unknown parameters r, θ, ρ. The results confirmed that the HRM depicted the hormetic data well, and revealed that the larger the intrinsic growth rate, the more likely is the occurrence of hormetic effects. Moreover, a comparison of the last data point and its simulated value in figure 2*a*, shows that the two large doses lead to redundancy.

## 4. Discussion

In order to formulate our novel discrete single species model with perturbation within each generation presented here, analytical and piecewise constant methods were used to extend the classic discrete Beverton–Holt and Ricker models, referred to as EBHM and HRM, respectively (see the electronic supplementary material). Based on the HRM, we revealed complex three-parameter spaces including: (1) the existence and stability of equilibria, shown in electronic supplementary material, tables S1 and S2 and (2) the occurrences of hormetic and paradoxical effects with respect to control parameters *θ* and *q,* shown in the electronic supplementary material, table S3. Moreover, the hormetic and paradoxical effects can be enhanced by multiple low-level stimulations within each generation, as shown in figure 6, i.e. the cumulative effects can significantly reduce the threshold levels, maximum responses and inhibition.

Theoretical analyses and numerical investigations confirm that hormesis is difficult to investigate, as it requires that three factors (growth, dose–response and dose timing response) can act together in a complex parameter space. The novel model that we have devised is capable of describing such hormesis and the phenomenon of ecological paradoxes. The results derived from the model show how interactions between intervention dose and dose timing and between dose–responses and intrinsic factors can model hormesis in toxicological experiments and ecological paradoxes, providing insights into their complex dynamics and a methodology for improved design and analysis of experiments, with wide-reaching implications for understanding hormetic effects.

Our study reveals two basic situations with hormetic effects and thus two routes to hormesis. One is the dual role of bioregulatory and compensation mechanisms in inducing new homeostatic states once the external stimulation occurs at the right time, even though external stimulation reduces the intrinsic growth rate (for example, when *r* < 2, i.e. the previous homeostatic state is stable). However, due to the variation in intraspecific regulatory factors and compensation mechanisms, the intrinsic reproductive capacity can be enhanced when the population density reaches a certain threshold level (i.e. the rapidly increasing *f*(*N*), as shown in figure 3*a*), to achieve a new high-level equilibrium that is much greater than the previous one, occurring with hormetic effects (figure 4*i–k*). The other situation results from the interaction between the intraspecific regulation factor, compensation mechanism and induced stability (the previous homeostatic state is unstable when *r >* 2) when catastrophic shifts may be realized and alternative high homeostatic states reached, also occurring with hormetic effects (figure 4*l–p*).

For ecotoxicological applications, our model helps to determine whether a low-dose results in hormetic effects, and to evaluate the effectiveness of a high-dose pesticide. Moreover, it provides an important cue for determining action thresholds and pest control strategies [35,36]. Recognition of hormetic-like biphasic dose–responses and dose timing responses and how they are produced are crucial for elucidating bioregulatory actions and their biomedical implications [18,37–40]. The main features shown in figures 4–6 and the complex parameter spaces accounting for the occurrence of the features listed in the electronic supplementary material, tables S1–S4 reveal why HPE phenomena are generalizable. Also, they explain why most toxicological experiments lack the capacity to assess possible hormetic dose–responses [4]. Our main results reveal that not only dose factors, but also the dose timing responses of interventions and population growth patterns are important determinants of hormetic effects. Therefore, the theory predicts that there may be as yet undetected hormetic effects in many practical circumstances, in addition to those involved in pest control and disease treatments.

The theoretical analyses revealed the close relationships between the internal growth process of the pest population and the dosage of pesticide application, as well as the dose timing response of the applications, i.e. the occurrence of hormetic and paradoxical effects are determined by the complex three-parameter spaces including the intrinsic growth rate, the dose–response and the dose timing response of pesticide implementations. Nevertheless, we found that the three parameters give the dynamic behaviour of the system in the form of a regular combination. The threshold values related to the combination rθ give the key conditions for the existence of equilibria, as shown in electronic supplementary material, table S1. However, the stability properties of the equilibria and occurrences of hormetic and paradoxical effects are determined by the threshold values of the combination $pe^{r}$ or r+lnp, as shown in electronic supplementary material, tables S2–S4. It is interesting that in a certain parameter space, the hormetic and paradoxical effects could occur whether the equilibrium is stable or unstable. Moreover, the critical values for the occurrence of hormetic and paradoxical effects related to the *q* and *θ* are quite different, i.e. for the fixed dose–response *q* the critical intrinsic growth is divided into four different intervals shown in electronic supplementary material, table S3, while only two critical intervals are separated for the *θ* shown in electronic supplementary material, table S4 which requires a large intrinsic growth rate. All these important relations among three crucial parameters can help us to distinguish the occurrence of negative effects of pesticide applications, and then help us to recognize hormesis in toxicological experiments and ecological paradoxes.

Importantly, the models and analytical techniques presented here not only provide a possibility for assessing the effectiveness of control interventions within two generations of discrete populations, but they can also be employed to fit field data with multiple pulse perturbations, as shown in figure 1, and to hormetic data, as shown in figure 2. Thus, given the growth characteristics of plant leaves, we fitted the growth rates of pests, and then were able to fit the field data from different regions. Moreover, such data fitting and parameter estimations can help to evaluate the parameter space related to the potential occurrence of hormetic and paradoxical effects.

In summary, in this paper, a novel re-formulation of the Ricker population equation showed how interactions between dose–responses, dose timing responses and intrinsic factors can model hormesis in toxicological experiments and ecological paradoxes, providing insights into their complex dynamics and a methodology for improved design and analysis of experiments, with implications for understanding hormetic effects in general. For ecological applications, the data fitting demonstrated the effectiveness of our framework to inform intervention strategies for species with discrete generations under multiple impulsive interventions.

We only focused on a single species model to reveal the effects of multiple factors on hormesis, and note that a key problem of how to construct a discrete model for multi-population interactions, based on methods similar to those developed here, remains a key theoretical challenge. Moreover, many factors such as environmental temperature, diet, pesticide or drug resistance, and random perturbation may also play important roles in inducing hormesis. Therefore, our next goal is to develop more practical mathematical models and carry out systematic research related to paradoxical and hormetic effects.

## Supplementary Material

Tang, Liang, Xiang, Xiao, Wang, Wu, Li & Cheke A general model of hormesis in biological systems and its application to pest management Supplementary Material

## Data accessibility

The field dataset are freely available from S.Y.

## References

[1] Thom R 1989 Structural stability and morphogenesis: an outline of a general theory of models. Reading, MA: Addison-Wesley.

[2] Scheffer M, Carpenter S, Foley JA, Folke C, Walker B 2001 Catastrophic shifts in ecosystems. Nature 413, 91–596. (10.1038/35098000) 11595939

[3] Cheke RA, Tang S, Tratalos JA 2014 Predator–prey population models of migrant insects with phase change*.* ICES J. Mar. Sci. 71, 2221–2230. (10.1093/icesjms/fst150)

[4] Calabrese EJ, Baldwin LA 2003 Toxicology rethinks its central belief. Nature 421, 691–692. (10.1038/421691a) 12610596

[5] Calabrese EJ 2002 Hormesis: changing view of the dose–response, a personal account of the history and current status. Mutat. Res. 511, 181–189. (10.1016/S1383-5742(02)00013-3) 12088716

[6] Erofeeva EA 2018 Hormesis and paradoxical effects of pea (*Pisum sativum* L.) parameters upon exposure to formaldehyde in a wide range of doses. Ecotoxicology 27, 569–577. (10.1007/s10646-018-1928-2) 29594892

[7] Lotka AJ 1932 The growth of mixed populations: two species competing for a common food supply. J. Wash. Acad. Sci. 22, 461–469. (10.1007/978-3-642-50151-7_12)

[8] Hutchinson GE 1961 The paradox of the plankton. Am. Nat. 95, 137–145. (10.2307/2458386)

[9] Record NR, Pershing AJ, Maps F 2014 Plankton post-paradox: reply to comment on ‘the paradox of ‘the paradox of the plankton’’ by Record *et al*. ICES J. Mar. Sci. 71, 296–298. (10.1093/icesjms/fst213)

[10] Volterra V 1926 Fluctuation in abundance of a species considered mathematically. Nature 118, 558–560. (10.4236/jamp.2015.37094)

[11] Engen S, Stenseth N-C 1984 An ecological paradox: a food type may become more rare in a diet as a consequence of being more abundant. Am. Nat. 124, 352–359. (10.1086/284278)

[12] Morse JG 1998 Agricultural implications of pesticide-induced hormesis of insects and mites. Hum. Exp. Toxicol. 17, 266–269. (10.1177/096032719801700510) 9663935

[13] Gaya A, Akle CA, Mudan S, Grange J 2015 The concept of hormesis in cancer therapy—is less more? Cureus 7, e261 (10.7759/cureus.261) 26180685 PMC4494563

[14] Pearce OMT, Läubli H, Verhagen A, Secrest P, Zhang J, Varki NM, Crocker PR, Bui JD, Varki A 2014 Inverse hormesis of cancer growth mediated by narrow ranges of tumor-directed antibodies. Proc. Natl Acad. Sci. USA 111, 5998–6003. (10.1073/pnas.1209067111) 24711415 PMC4000847

[15] Edward J, Staudenmayer JW, Stanek EJ 3rd, Hoffmann GR 2006 Hormesis outperforms threshold model in national cancer institute antitumor drug screening database. Toxicol. Sci. 94, 368–378. (10.1093/toxsci/kfl098) 16950854

[16] Dattilo S et al 2015 Heat shock proteins and hormesis in the diagnosis and treatment of neurodegenerative diseases. Immun. Ageing 12, 20 (10.1186/s12979-015-0046-8) 26543490 PMC4634585

[17] Hayes DP 2008 Adverse effects of nutritional inadequacy and excess: a hormetic model. Am. J. Clin. Nutr. 88, 578S–581S. (10.1186/1476-511X-7-27) 18689405

[18] Erofeeva EA 2014 Hormesis and paradoxical effects of wheat seedling (*Triticum aestivum* L.) parameters upon exposure to different pollutants in a wide range of doses. Dose-Response 12, 121–135. (10.2203/dose-response.13-017.erofeeva) 24659937 PMC3960958

[19] Cohen E 2006 Pesticide-mediated homeostatic modulation in arthropods. Pestic. Biochem. Physiol. 85, 21–27. (10.1016/j.pestbp.2005.09.002)

[20] Guedes RNC, Cutler GC 2014 Insecticide-induced hormesis and arthropod pest management. Pest Manag. Sci. 70, 690–697. (10.1002/ps.3669) 24155227

[21] Matsuoka T, Seno H 2008 Ecological balance in the native population dynamics may cause the paradox of pest control with harvesting. J. Theor. Biol. 252, 87–97. (10.1016/j.jtbi.2008.01.024) 18329048

[22] Seno H 2008 A paradox in discrete single species population dynamics with harvesting/thinning. Math. Biosci. 214, 63–69. (10.1016/j.mbs.2008.06.004) 18602928

[23] Cid B, Hilker FM, Liz E 2014 Harvest timing and its population dynamic consequences in a discrete single-species model. Math. Biosci. 248, 78–87. (10.1016/j.mbs.2013.12.003) 24361496

[24] Ricker WE 1954 Stock and recruitment. J. Fisheries Res. Brd. Canada 11, 559–623. (10.1139/f54-039)

[25] Carelli G, Lavicoli I 2002 Defining hormesis: the necessary tool to clarify experimentally the dose–response relationship. Hum. Exp. Toxicol. 21, 103–104. (10.1191/0960327102ht219oa) 12102492

[26] Liess M, Foit K, Becker A, Hassold E, Dolciotti I, Kattwinkel M, Duquesne S 2013 Culmination of low-dose pesticide effects. Environ. Sci. Technol. 47, 8862–8868. (10.1021/es401346d) 23859631 PMC3781603

[27] May RM 1975 Biological populations obeying difference equations: stable points, stable cycles, and chaos. J. Theor. Biol. 51, 511–524. (10.1016/0022-5193(75)90078-8) 1142800

[28] May RM 1976 Simple mathematical models with very complicated dynamics. Nature 261, 459–467. (10.1038/261459a0) 934280

[29] May RM, Oster GF 1976 Bifurcations and dynamic complexity in simple ecological models. Am. Nat. 110, 573–599. (10.1086/283092)

[30] May RM 1977 Thresholds and breakpoints in ecosystems with a multiplicity of stable states. Nature 269, 471–477. (10.1038/269471a0)

[31] Abbas T, Nadeem MA, Tanveer A, Zohaib A, Rasool T 2015 Glyphosate hormesis increases growth and yield of chickpea (*Cicer arietinum* l.). Pak. J. Weed Sci. Res. 21, 533–542.

[32] Guedes NMP, Tolledo J, Corrêa AS, Guedes RNC 2010 Insecticide-induced hormesis in an insecticide-resistant strain of the maize weevil, *Sitophilus zeamais**.* J. Appl. Entomol. 134, 142–148. (10.1111/j.1439-0418.2009.01462.x)

[33] National Cotton Council. Growth and development of a cotton plant. See http://www.cotton.org/tech/ace/growth-and-development.cfm. (accessed 1 March 2019)

[34] Beverton RJ, Holt SJ 1956 The theory of fishing. In Sea fisheries; their investigation in the United Kingdom (ed. Graham M), pp. 372–441. London, UK: Edward Arnold.

[35] Tang S, Xiao Y, Cheke RA 2008 Multiple attractors of host–parasitoid models with integrated pest management strategies: eradication, persistence and outbreak. Theoret. Pop. Biol. 73, 181–197. (10.1016/j.tpb.2007.12.001) 18215410

[36] Tang S, Liang J, Tan Y, Cheke RA 2013 Threshold conditions for integrated pest management models with pesticides that have residual effects. J. Math. Biol. 66, 1–35. (10.1007/s00285-011-0501-x) 22205243

[37] Calabrese EJ, Baldwin LA 2001 The frequency of U-shaped dose responses in the toxicological literature. Toxicol. Sci. 62, 330–338. (10.1093/toxsci/62.2.330) 11452146

[38] Scheffer M, Hosper SH, Meijer ML, Moss B, Jeppesen E 1993 Alternative equilibria in shallow lakes. Trends Ecol. Evol. 8, 275–279. (10.1016/0169-5347(93)90254-M) 21236168

[39] Wetzel WC, Kharouba HM, Robinson M, Holyoak M, Karban R 1993 Variability in plant nutrients reduces insect herbivore performance. Nature 539, 425–427. (10.1038/nature20140) 27749815

[40] Calabrese EJ 2009 Getting the dose response wrong: why hormesis became marginalized and the threshold model accepted. Arch. Toxicol. 83, 227–247. (10.1007/s00204-009-0411-5) 19234688

