# Supplementary material for: A general model of hormesis in biological systems and its application to pest management
Source: J R Soc Interface. 2019 Aug 21;16(157):20190468. doi: 10.1098/rsif.2019.0468 (PMC6731494; doi:10.1098/rsif.2019.0468)
Supplement: Tang, Liang, Xiang, Xiao, Wang, Wu, Li & Cheke A general model of hormesis in biological systems and its application to pest management Supplementary Material [file rsif20190468supp1.pdf]

Tang, Liang, Xiang, Xiao, Wang, Wu, Li & Cheke

A general model of hormesis in biological systems and its application to pest management Supplementary Material

Contents

1 Model formulation 2

1.1 The extended Beverton-Holt model (EBHM) 2

1.2 The hormesis Ricker model (HRM) 4

2 Dynamics of HRM with $\theta \in (0, 1)$ 6

2.1 The existence of equilibrium N^* 6

3 Positive equilibrium and its stability 15

3.1 The local stability of equilibrium N^* 27

4 Hormesis and paradox with respect to parameters p and θ 35

5 Multiple control actions within each generation 45

6 Some important definitions related to the Lambert W function 46

1. Model formulation

In order to formulate the novel discrete single species model with perturbation within each generation, analytical and piecewise constant methods were used to extend the classic discrete Beverton-Holt and Ricker models [1, 2, 3].

1.1. The extended Beverton-Holt model (EBHM)

Consider the following Logistic equation

$$\frac{dN(t)}{dt} = rN(t)\left[1 - \frac{N(t)}{K}\right] \tag{S1.1}$$

with each integer interval $t \in [n, n+1]$. Although model (S1.1) can describe the growth of many kinds of populations, including tumor cells and pests, as an example for modelling control tactics we only consider a pest population in the following. Without loss of generality, assuming that there exists a positive constant $\theta \in [0, 1]$ such that a chemical control tactic was applied at $n + \theta$ and a proportion of the pest was killed, denoted by q (characterization of pesticide or drug dose or effectiveness) so that $p = 1 - q$ is the survival rate after the pesticide has been sprayed.

The analytical solution of the logistic equation for any initial value N_n is given by

$$N(t) = \frac{KN_n}{N_n + (K - N_n)e^{-r(t-n)}}, t \in (n, n+\theta] \tag{S1.2}$$

and by denoting $c = \frac{K-N_n}{N_n}$, the above equation becomes

$$N(t) = \frac{K}{1 + ce^{-r(t-n)}}, t \in (n, n+\theta]. \tag{S1.3}$$

Therefore, at time point $n + \theta$ we have

$$N_\theta \doteq N(n+\theta) = \frac{K}{1 + ce^{-r\theta}} \tag{S1.4}$$

and

$$N_\theta^+ \doteq N(n+\theta^+) = \frac{pK}{1+ce^{-r\theta}} = pN(n+\theta) = pN_\theta. \tag{S1.5}$$

Solving equation (S1.1) in the interval $(n+\theta, n+1]$ with initial value $N(n+\theta^+)$, yields

$$N(t) = \frac{K}{1+c_\theta e^{-r(t-n-\theta)}} \tag{S1.6}$$

with $c_\theta = \frac{K-N_\theta^+}{N_\theta^+}$.

Thus,

$$N_{n+1} \doteq N(n+1) = \frac{K}{1+c_\theta e^{-r(1-\theta)}} = \frac{KR}{R+c_\theta e^{-r\theta}} \tag{S1.7}$$

with $R = e^r$, and substituting c_θ and c into the above equation, yields

$$N_{n+1} = \frac{pKRN_n}{\left[p(R-R^\theta)+R^\theta-1\right]N_n+K}. \tag{S1.8}$$

This is the EBHM with instantaneous chemical control within each generation [6]. The two special cases can be obtained by letting $\theta = 0$ and $\theta = 1$.

There are two steady states for model (S1.8), which can be calculated as

$$N_0^* = 0, \quad N^* = \frac{K(pR-1)}{pR-1+R^\theta(1-p)}.$$

By simple calculation, for the positive equilibrium N^*, we have

$$\frac{dN^*}{dp} = \frac{KR^\theta(R-1)}{[pR-1+R^\theta(1-p)]^2} > 0 \cong \frac{dN^*}{dq} < 0 \tag{S1.9}$$

and

$$\frac{dN^*}{d\theta} = \frac{K(Rp-1)R^\theta \ln(R)(p-1)}{[pR-1+R^\theta(1-p)]^2} < 0. \tag{S1.10}$$

These derivations of N^* with respect to p and θ indicate that the later the spraying of pesticide and the larger the killing rate, the more effective is the control in relation to the stable population level, and these results show that the paradox and hormesis could not occur for the Beverton-Holt model when the chemical control action is applied within a discrete generation.

1.2. The hormesis Ricker model (HRM)

The piecewise constant method is employed in this subsection to derive the discrete Ricker model with instantaneous perturbations within each generation, i.e., we consider the following piecewise single species model

$$\frac{dN(t)}{dt} = rN(t)\left[1 - \frac{N_n}{K}\right],\ t \in (n, n+\theta] \tag{S1.11}$$

with

$$N_\theta^+ \doteq N(n+\theta^+) = pN(n+\theta)$$

and

$$\frac{dN(t)}{dt} = rN(t)\left[1 - \frac{N_\theta^+}{K}\right],\ t \in (n+\theta, n+1]. \tag{S1.12}$$

Solving Eq.(S1.11), yields

$$N(t) = N_n \exp\left[r\left(1 - \frac{N_n}{K}\right)(t-n)\right],\ t \in (n, n+\theta]$$

and

$$N_\theta \doteq N(n+\theta) = N_n \exp\left[r\left(1 - \frac{N_n}{K}\right)\theta\right],\ N_\theta^+ = pN_n \exp\left[r\left(1 - \frac{N_n}{K}\right)\theta\right].$$

Solving Eq.(S1.12), yields

$$N(t) = N_\theta^+ \exp\left[r\left(1 - \frac{N_\theta^+}{K}\right)(t-n-\theta)\right],\ t \in (n+\theta, n+1],$$

which indicates that

$$\begin{aligned} N_{n+1} &= pN_n \exp\left[r\left(1 - \frac{N_n}{K}\right)\theta + r\left(1 - \frac{pN_n}{K}\exp\left(r\left(1 - \frac{N_n}{K}\right)\theta\right)\right)(1-\theta)\right] \\ &= pN_n \exp\left[r\left(1 - \frac{N_n}{K}\left(\theta + (1-\theta)p\exp\left(r\left(1 - \frac{N_n}{K}\right)\theta\right)\right)\right)\right]. \end{aligned} \tag{S1.13}$$

This is the HRM with instantaneous chemical control within each generation [1].

A typical feature of models $EBHM$ and HRM, is that the control measure is applied instantaneously at time point $n + \theta$ within two successive generations of the population, an impulsive control strategy known as pulsed chemotherapy for tumour control [4] and as pulsed application of pesticide or pulsed release of natural enemies for pest control [5]. Thus, the parameter θ is referred to as the dose timing response. The parameter q (i.e. the pesticide instant killing rate in Math.) is characterized by the pesticide efficacy of a particular dose applied at time point $n+\theta$ [4, 5]. Thus, the parameter q, for convenience, is referred to as the dose response in the main text.

Note that the carrying capacity parameter K does not influence the dynamics of models $EBHM$ and HRM [1]. Therefore, one of our main purposes is to reveal the effects of population dynamics (intrinsic growth rate r here), the dose response (parameter q here) and the dose timing response (parameter θ here) on the occurrence of paradoxical and hormetic effects. Of course other factors such as environmental temperature, diet and pesticide or drug resistance, may also play important roles in inducing hormesis. Through detailed theoretical analyses, we provide here the complete three-parameter spaces for the occurrence of the above phenomena, which provide guidance on experimental design and determine potential risks.

2. Dynamics of HRM with $\theta \in (0,1)$

The non-trivial equilibrium N^* of model (S1.13) satisfies the following equation

$$p\exp\left[r\left(1-\frac{N^*}{K}\left(\theta+(1-\theta)p\exp\left(r\left(1-\frac{N^*}{K}\right)\theta\right)\right)\right)\right]=1 \qquad \text{(S2.1)}$$

i.e., we have

$$r+\ln(p)=\frac{rN^*}{K}\left[\theta+(1-\theta)p\exp\left(r\left(1-\frac{N^*}{K}\right)\theta\right)\right] \qquad \text{(S2.2)}$$

and denote the function $F(N^*)$ as follows

$$F(N^*)=p\exp\left[r\left(1-\frac{N^*}{K}\left(\theta+(1-\theta)p\exp\left(r\left(1-\frac{N^*}{K}\right)\theta\right)\right)\right)\right]-1. \qquad \text{(S2.3)}$$

2.1. The existence of equilibrium N^*

In order to discuss the existence of the positive equilibria of model (S1.13), we only need to address the existence of positive roots of equation $F(N)=0$ with

$$F(N)=p\exp\left[r\left(1-\frac{N}{K}\left(\theta+(1-\theta)p\exp\left(r\left(1-\frac{N}{K}\right)\theta\right)\right)\right)\right]-1,\ N>0.$$

To do this, we denote

$$N_1=\frac{K}{r\theta}\left[1-W(0,A)\right],\ N_2=\frac{K}{r\theta}\left[1-W(-1,A)\right]$$

with $A=\frac{\theta}{(\theta-1)pe^{r\theta-1}}$, please see more details from the last section of this SI for the definition of the Lambert W function. Obviously, we have $A<0$ due to $\theta\in(0,1)$ and the relations between A and $-e^{-1}$ will play a key role in determining the sign of $F'(N)$ and the number of positive roots of the

equation $F(N)=0$. Note that, according to the definition of the Lambert W function, $A \geq -e^{-1}$ ensures that N_1 and N_2 are well defined, and $N_1 = N_2 = \frac{2K}{r\theta}$ at $A=-e^{-1}$. Thus, at first we examine the equation

$$A = \frac{\theta}{(\theta-1)pe^{r\theta-1}} = -e^{-1} \Leftrightarrow \theta e = (1-\theta)pe^{r\theta-1}.$$

Solving the above equation with respect to r, yields

$$r = \frac{1}{\theta}\left[\ln\left(\frac{\theta e}{(1-\theta)p}\right)+1\right] = \frac{1}{\theta}\left[\ln\left(\frac{\theta}{(1-\theta)p}\right)+2\right] \doteq r_c,$$

which can help us to determine the relations between A and $-e^{-1}$, i.e., $r > r_c$ indicates that $A > -e^{-1}$. Note that r_c could be less than zero; if so, $A > -e^{-1}$ holds true for all $r > 0$. Thus, unless otherwise specified we always assume that $A \geq -e^{-1}$ when $W(0,A)$ and $W(-1,A)$ are used and considered. For convenience, we denote the three-parameter space satisfying $A=-e^{-1}$ as

$$\Omega^{A}_{(r,p,\theta)} = \left\{(r,p,\theta)\,\middle|\, r = \frac{1}{\theta}\left[\ln\left(\frac{\theta}{(1-\theta)p}\right)+2\right]\right\} \tag{S2.4}$$

and consequently we denote the parameter sets $\Omega^{A>0}_{(r,p,\theta)}$ (or $\Omega^{A\geq 0}_{(r,p,\theta)}$) and $\Omega^{A<0}_{(r,p,\theta)}$ as the parameter spaces for $A > -e^{-1}$ (or $A \geq -e^{-1}$) and $A < -e^{-1}$, respectively. Unless otherwise stated, similar notation has been used throughout.

Further, we denote $A_1 = 1 - W(0,A)$ and $A_2 = 1 - W(-1,A)$, and by simple calculations we have

$$F(0) = pe^{r} - 1,\ F(K/(r\theta)) = pe^{r-1}e^{\frac{1}{A}} - 1,$$

$$F(N_1) = pe^{r}e^{\frac{(1-W[0,A])^2}{W[0,A]}} - 1,\ F(N_2) = pe^{r}e^{\frac{(1-W[-1,A])^2}{W[-1,A]}} - 1$$

and

$$F(K) = pe^{r(1-\theta)(1-p)} - 1 < F(0),\ F(+\infty) = -1.$$

Moreover, we can prove that $F(N_i) < F(0)(i = 1, 2)$ provided that $-e^{-1} \leq A$ and $F(0) \geq 0$ provided that a positive equilibrium exists for model (S1.13).

Now we first discuss the signs of both functions $F(N_1)$ and $F(N_2)$, so for $k = 0$ and -1 we let

$$pe^r e^{\frac{(1-W[k,A])^2}{W[k,A]}} - 1 = 0$$

i.e.,

$$\frac{(1 - W[k, A])^2}{W[k, A]} = -r - \ln(p)$$

which indicates that

$$W(k, A) = \frac{a}{2} + 1 \pm \frac{1}{2}\sqrt{(a + 2)^2 - 4} = \frac{a}{2} + 1 \pm \sqrt{\left(\frac{a}{2} + 1\right)^2 - 1}$$

with $a = -r - \ln(p)$. Note that $pe^r > 1$ (i.e., $F(0) > 0$) indicates that $a < 0$. Therefore, in order to ensure that $W(k, A)$ are well defined for $k = 0, -1$, we must have $a \leq -4$ with $W(k, A) = -1$ at $a = -4$. This means that $r + \ln(p) \geq 4$, i.e., we have $pe^r \geq e^4$. For $k = 0$, we must have

$$\frac{a}{2} + 1 + \sqrt{\left(\frac{a}{2} + 1\right)^2 - 1} \geq -1 \text{ for } a \leq -4,$$

which holds true naturally for $a \leq -4$. However, for $k = -1$, we must have

$$\frac{a}{2} + 1 - \sqrt{\left(\frac{a}{2} + 1\right)^2 - 1} \leq -1 \text{ for } a \leq -4,$$

which also holds true naturally for $a \leq -4$.

Furthermore according to the definition of the Lambert W function we have

$$\left[\frac{a}{2} + 1 \pm \sqrt{\left(\frac{a}{2} + 1\right)^2 - 1}\right] e^{\left[\frac{a}{2}+1\pm\sqrt{\left(\frac{a}{2}+1\right)^2-1}\right]} = A = \frac{\theta}{(\theta - 1)pe^{r\theta-1}} \quad \text{(S2.5)}$$

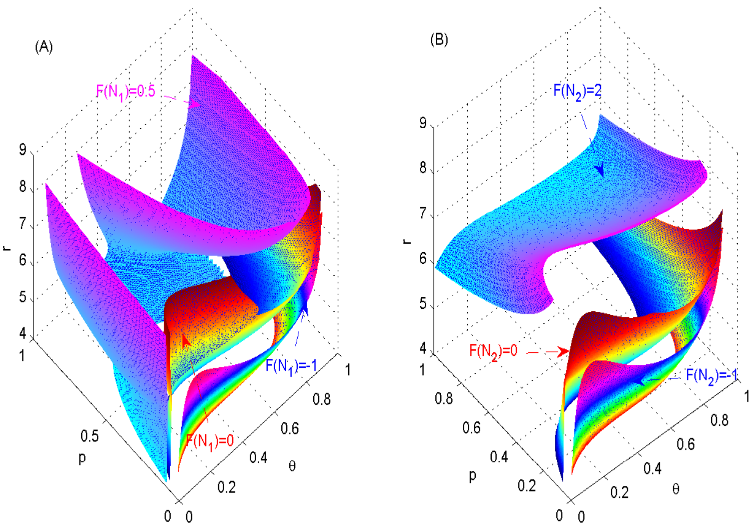

Figure S.1: Illustration of the signs of $F(N_1)$ and $F(N_2)$ in three-parameter space which satisfies $r + \ln(p) \geq 4$. Three curves with $F(N_1) = 0.5, F(N_1) = 0$ and $F(N_1) = -1$ are shown in (A), and three curves with $F(N_2) = 2, F(N_2) = 0$ and $F(N_2) = -1$ are shown in (B).

which depict the relations among three key parameters r, p and θ such that $F(N_1) = 0$ and $F(N_2) = 0$, respectively, denoted by $\Omega^{F(N_1)}_{(r,p,\theta)}, \Omega^{F(N_2)}_{(r,p,\theta)}$ with $r + \ln(p) \geq 4$. Therefore, based on the equations given by (S2.5), the three-parameter space which determines the signs of the functions $F(N_1)$ and $F(N_2)$ can be implicitly given, as shown in Fig.S.1. This shows that the parameter spaces related to the signs of both functions are quite complex. Furthermore, we have the following results.

Lemma 1 If $-e^{-1} \leq A$, then $F(N_i) < F(0),\ i = 1, 2$.

Proof. It follows from $F'(N_i) = 0$ that we have $h(N_i) = l(N_i),\ i = 1, 2$. Thus

$$\begin{aligned} g(N_i) &= r\left[1 - \tfrac{N_i}{K}(\theta + h(N_i))\right] \\ &= r\left[1 - \tfrac{N_i}{K}(\theta + l(N_i))\right] \\ &= r\left[1 - \tfrac{N_i}{K}\left(\theta + \tfrac{\theta}{\frac{r\theta N_i}{K} - 1}\right)\right] \\ &= r\left[1 - \tfrac{r\theta^2 N_i^2}{K(r\theta N_i - K)}\right]. \end{aligned} \quad \text{(S2.6)}$$

According to the expression of $N_i (i = 1, 2)$, we have

$$\begin{aligned} r\theta N_1 - K &= K(1 - W(0, A)) - K \\ &= -KW(0, A) > 0, \\ r\theta N_2 - K &= -KW(-1, A) > 0. \end{aligned}$$

Therefore, $g(N_i) < r(i = 1, 2)$. All these results confirm that

$$F(N_i) = pe^{g(N_i)} - 1 < pe^r - 1 = F(0),\ i = 1, 2.$$

This completes the proof of Lemma 1.

For convenience, denote

$$h(N) = (1 - \theta)p \exp\left[r\left(1 - \frac{N}{K}\right)\theta\right], \quad \text{(S2.7)}$$

$$g(N) = r\left[1 - \frac{N}{K}(\theta + h(N))\right], \tag{S2.8}$$

and

$$l(N) = \frac{\theta}{\frac{r\theta N}{K} - 1}.$$

Lemma 2 If a positive equilibrium exists for model (S1.13) , then $pe^r > 1$ (i.e., $F(0) > 0$).

Proof. If follows from the definition of the function $F(N)$ and the above notation that

$$F(N) = pe^{g(N)} - 1, \text{and } f(N) \doteq pNe^{g(N)}, \; N \geq 0.$$

Obviously the zero root always exists for the equation $f(N) = N$, denoted by N_0^*. If N^* is the positive root of equation $F(N) = 0$, which satisfies

$$pe^{g(N^*)} = 1. \tag{S2.9}$$

It follows from

$$g(N^*) = r\left[1 - \frac{N^*}{K}(\theta + h(N^*))\right] < r$$

that

$$pe^r > pe^{g(N^*)} = 1, \text{ i.e., } \; F(0) = pe^r - 1 > 0.$$

This completes the proof of Lemma 2.

Based on the above results, we first discuss the monotonicity of function $F(N)$, and then address the positive roots of the equation $F(N) = 0$. For the monotonicity of function $F(N)$ we have the following main results.

Theorem 1 If $1 - \frac{r\theta}{K}N > 0$, then $F'(N) < 0$ for all $N \in \left(0, \frac{K}{r\theta}\right)$; if $1 - \frac{r\theta}{K}N < 0$ and $A < -e^{-1}$, then $F'(N) < 0$ for all $N \in \left(\frac{K}{r\theta}, +\infty\right)$; if $1 - \frac{r\theta}{K}N < 0$

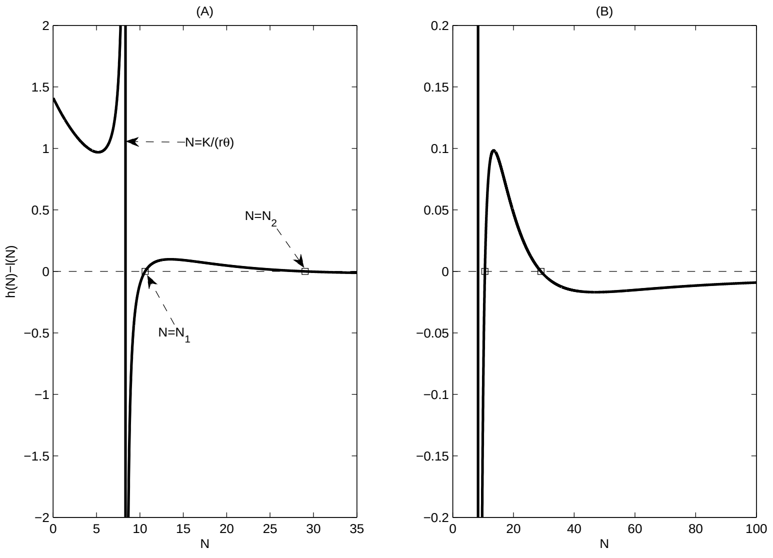

Figure S.2: Illustration of monotonicity of function $F(N)$, where $h(N)$ and $l(N)$ are defined in the text.

and $-e^{-1} \leq A < 0$, then $F'(N) < 0$ for all $N \in \left(\frac{K}{r\theta}, N_1\right) \cup (N_2, +\infty)$ and $F'(N) \geq 0$ for all $N \in [N_1, N_2]$.

Proof. Taking the derivative of $F(N)$ with respect to N, yields

$$\begin{aligned} F'(N) &= p\exp[g(N)]g'(N) \\ &= p\exp[g(N)]\left[-\tfrac{r}{K}(\theta + h(N) + xh'(N))\right] \\ &= p\exp[g(N)]\left[-\tfrac{r}{K}\left(\theta + h(N) + N\left(-\tfrac{r\theta}{K}h(N)\right)\right)\right] \quad \text{(S2.10)} \\ &= -\tfrac{r}{K}p\exp[g(N)]\left[\theta + \left(1 - \tfrac{r\theta}{K}N\right)h(N)\right] \\ &= -\tfrac{r}{K}p\exp[g(N)]\left(1 - \tfrac{r\theta}{K}N\right)[h(N) - l(N)]\,. \end{aligned}$$

To determine the sign of $F'(N)$, the signs of two parts, i.e., $1 - \frac{r\theta}{K}N$ and $h(N) - l(N)$ play key roles. Note that, if $1 - \frac{r\theta}{K}N > 0$, then $\theta + \left(1 - \frac{r\theta}{K}N\right)h(N) > 0$, and consequently $F'(N) < 0$. Therefore, we will focus on the case $1 - \frac{r\theta}{K}N < 0$ (i.e., $N > \frac{K}{r\theta}$) in the following.

Letting $F'(N) = 0$, we have $h(N) = l(N)$, that is

$$(1-\theta)p\exp\left[r\left(1 - \frac{N}{K}\right)\theta\right] = \frac{\theta}{\frac{r\theta N}{K} - 1} \quad \text{(S2.11)}$$

or

$$\left(1 - \frac{r\theta}{K}N\right)e^{1 - \frac{r\theta}{K}N} = A.$$

Note that $l(N) > 0$ for all $N > \frac{K}{r\theta}$, and $\lim_{N \to +\frac{K}{r\theta}} l(N) = +\infty$, which means that

$$\lim_{N \to +\frac{K}{r\theta}} (h(N) - l(N)) < 0 \text{ and } \lim_{N \to +\infty} (h(N) - l(N)) = 0.$$

Moreover, by a simple calculation, we have

$$h'(N) - l'(N) = \frac{r\theta}{K}\left[h(N) - \frac{l(N)}{\frac{r\theta N}{K} - 1}\right],$$

and it follows from $N_2 \geq \frac{2K}{r\theta}$ that $h'(N_2) - l'(N_2) \geq 0$. Further, if $A \geq -e^{-1}$, then it is interesting that equation (S2.11) can be solved analytically by using the definition of the Lambert W function, i.e., the two positive roots are given by N_1 and N_2. All these results confirm that the curve of function $h(N) - l(N)$ should be as those given in Fig.S.2 (here $A > -e^{-1}$ holds true).

Therefore, based on the properties of the Lambert W function and the relations between A and $-e^{-1}$, we discuss the following sub-cases for the sign of the function $F'(N)$.

Case a. $A < -e^{-1}$, i.e., equation (S2.11) does not have any positive root.

It follows from the properties of the function xe^x that

$$\left(1 - \frac{r\theta}{K}N\right) e^{1-\frac{r\theta}{K}N} > A \text{ for } N > \frac{K}{r\theta},$$

this indicates that

$$\theta + \left(1 - \frac{r\theta}{K}N\right) h(N) > 0, \text{ when } 1 - \frac{r\theta}{K}N < 0 \text{ for } N > \frac{K}{r\theta}.$$

Therefore, we have $F'(N) < 0$ for $N > \frac{K}{r\theta}$ and $A < -e^{-1}$.

Case b. $-e^{-1} \leq A < 0$, i.e., for equation (S2.11) there exist two positive roots N_1 and N_2.

It follows from

$$-1 < W[0, A] < 0 \text{ and } W[-1, A] < -1$$

that we have

$$\frac{K}{r\theta} < N_1 \leq \frac{2K}{r\theta} \leq N_2.$$

Thus

$$F'(N) \geq 0 \;\Leftrightarrow\; h(N) \geq l(N) \;\Leftrightarrow\; N \in [N_1, N_2]$$

and

$$F'(N) < 0 \;\Leftrightarrow\; h(N) < l(N) \;\Leftrightarrow\; N \in \left(\frac{K}{r\theta}, N_1\right) \cup (N_2, +\infty)\,.$$

This completes the proof.

3. Positive equilibrium and its stability

According to Lemma 2, the necessary condition for the existence of the positive equilibrium is $pe^r > 1$ (i.e., $F(0) > 0$). Thus, we assume that $pe^r > 1$ (i.e., $r + \ln(p) > 0$ or $a < 0$) in the following. Based on the monotonicity of the function $F(N)$ the following cases for the existence of positive roots of the function $F(N) = 0$ are discussed below.

Case A. $A < -e^{-1} \Leftrightarrow r\theta < r_c\theta = \ln\left(\frac{\theta}{(1-\theta)p}\right) + 2$, i.e., equation (S2.11) does not have any positive root.

It follows from Theorem 1 that $F'(N) < 0$ for all $N \in (0, +\infty)$. Further, according $F(0) > 0$ and $\lim\limits_{N \to +\infty} F(N) < 0$, we can see that for the equation $F(N) = 0$, there exists a unique positive root in $(0, +\infty)$. Moreover, if $F(K) \leq 0$, then the unique positive root must lie in $(0, K]$.

Case B. $-e^{-1} \leq A < 0 \Leftrightarrow r\theta \geq r_c\theta$, i.e., for equation (S2.11) there exist two positive roots N_1 and N_2.

It follows from Theorem 1 that $F(N)$ is decreasing for $N \in (0, N_1) \cup (N_2, +\infty)$, and increasing for $N \in [N_1, N_2]$, as shown in Fig.S.3. Therefore, if $F(N_1) > 0$, then equation $F(N) = 0$ has only one root which lies in

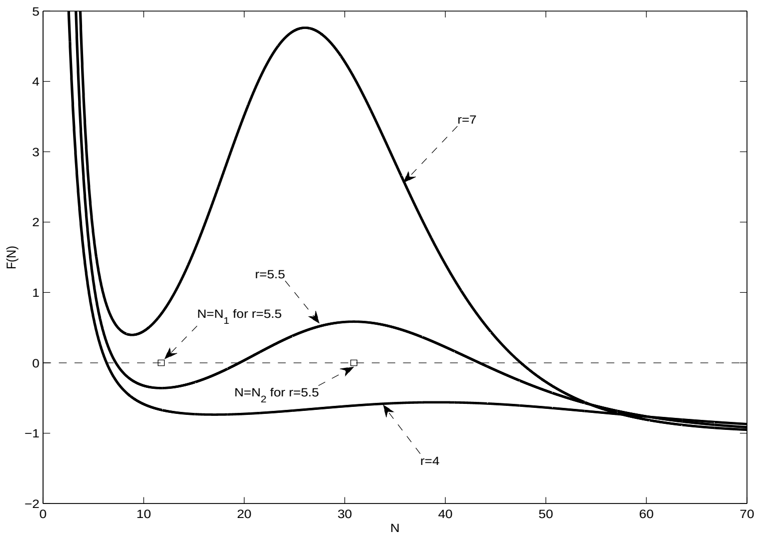

Figure S.3: Illustration of the existence of the positive equilibrium for Case B, where $F(N)$ is defined in the main text and the other parameters are fixed as: $\theta = 0.1, p = 0.8, K = 5$.

$(N_2, +\infty)$; if $F(N_2) < 0$, then equation $F(N) = 0$ has only one root which lies in $(0, N_1)$; in particular, if $F(N_1) = 0$, then equation $F(N) = 0$ has two roots in $N \in (0, +\infty)$, where one root is $N = N_1$, and the other root is in $(N_2, +\infty)$; if $F(N_2) = 0$, then equation $F(N) = 0$ has two roots in $N \in (0, +\infty)$, where one root is $N = N_2$ and the other is in $(0, N_1)$; if $F(N_1) < 0$ and $F(N_2) > 0$, then equation $F(N) = 0$ has three roots in $N \in (0, +\infty)$, where the first root is in $(0, N_1)$, the second root is in (N_1, N_2), and the last one is in $(N_2, +\infty)$. Furthermore, if the positive roots exist, then we denote them as $N_1^*,\ N_2^*,\ N_3^*$, respectively, where $N_1^* \in (0, N_1),\ N_2^* \in (N_1, N_2),\ N_3^* \in (N_2, +\infty)$.

The relations among $N_i^*(i = 1, 2, 3)$ and K are quite important to show the effects of control measures on outbreaks of the pest population, and to depict the occurrences of a paradox and hormesis due to chemical applications. According to the value of $r\theta$, we consider the following several cases.

Case B1. $r_c\theta \leq r\theta < 1$

For this case we have the following relations:

$$K < \frac{K}{r\theta} < N_1 \leq \frac{2K}{r\theta} \leq N_2.$$

Based on the above inequalities, we can obtain that if $F(N_1) > 0$, then equation $F(N) = 0$ has only one root N_3^* with $N_3^* > N_2 > K$; if $F(K) < 0$ and $F(N_2) < 0$, then equation $F(N) = 0$ has only one root N_1^* with $N_1^* < K$; if $F(K) > 0$ and $F(N_2) < 0$, then equation $F(N) = 0$ has only one root N_1^* with $K < N_1^* < N_1$; if $F(K) < 0$ and $F(N_2) > 0$, then equation $F(N) = 0$ has three roots $N_1^*,\ N_2^*,\ N_3^*$ with $N_1^* < K < N_1 < N_2^* < N_2 < N_3^*$; if $F(K) > 0$, $F(N_1) < 0$ and $F(N_2) > 0$, then equation $F(N) = 0$ has three roots $N_1^*,\ N_2^*,\ N_3^*$ with $K < N_1^* < N_1 < N_2^* < N_2 < N_3^*$.

Note that if $p = 0$ and $\theta = 0$, then $F(K) = F(0)$. Thus, for this very special case the carrying capacity K is a unique positive equilibrium of the model. However, the values of positive equilibria, N_1^*, N_2^*, N_3^*, could be largely changed once a single pesticide application has been applied within the generation. The formula

$$F(K) = pe^{r(1-\theta)(1-p)} - 1$$

clearly shows how the dose timing (i.e., θ) and the dose response (i.e., p) of the pesticide applications affect the sign of $F(K)$. To address this in more detail, we have the following results.

Lemma 3 Assume that $r > 1$ and $p \in (p_c^2, p_c^1)$, then there exists a unique threshold parameter $\theta_c \in (0, 1)$ such that $F(K) > 0$ for $\theta < \theta_c$ and $F(K) < 0$ for $\theta > \theta_c$, where $p_c^1 = 1$ and $p_c^2 = -\frac{1}{r}W(0, -re^{-r})$.

Proof. Letting

$$F(K) = pe^{r(1-\theta)(1-p)} - 1 = 0$$

and solving it with respect to θ, yields

$$\theta_c = \frac{r(1-p) + \ln(p)}{r(1-p)}.$$

It is easy to see that $\theta_c < 1$. To ensure $\theta_c \in (0, 1)$, we first consider the equation $r(1-p)+\ln(p) = 0$ with respect to p. Rearranging $r(1-p)+\ln(p) = 0$, yields the following equation

$$pe^{r(1-p)} = 1, \quad \text{i.e.,} \quad -rpe^{-rp} = -re^{-r}.$$

Solving the above equation with respect to p and according to $p \in [0, 1]$, yields two roots

$$p_c^1 = 1, \text{ and } p_c^2 = -\frac{1}{r}W(0, -re^{-r}).$$

Similarly, according to the properties of the function $-re^{-r}$ and the Lambert W function, we see that $p_c^2 = 1$ for $r \in (0,1]$ or $r = -W(0,-re^{-r})$ for $r \in (0,1]$. Moreover, by a simple calculation for $r \neq 1$, we have

$$\frac{dp_c^2}{dr} = \frac{W(0,-re^{-r})\,[r + W(0,-re^{-r})]}{r^2\,[1 + W(0,-re^{-r})]},$$

which indicates that $\frac{dp_c^2}{dr} < 0$ for $r > 1$, and consequently, we have $p_c^2 < 1$ for $r > 1$. Moreover, the function $r(1-p) + \ln(p)$ with respect to p reaches its maximal value at $p = \frac{1}{r}$ with $r > 1$. These results confirm that the p_c^2 is well defined for all given $r > 1$, and consequently, the threshold θ_c given in the above is also well defined for all $p \in (p_c^2, p_c^1)$ and $r > 1$. This completes the proof.

Lemma 3 reveals that for a certain range of instant killing rate and a relatively large intrinsic growth rate r (i.e., $r > 1$ here), the different timings of pesticide applications may either successfully suppress the density of the pest population below the carrying capacity or cause pest population outbreaks and even resurgence with a density larger than the carrying capacity K, with the occurrence of hormetic and paradoxical effects.

Note that we can also solve r from the equation $pe^{r(1-\theta)(1-p)} - 1 = 0$, yielding

$$r_c^4 = \frac{-\ln(p)}{(1-p)(1-\theta)}.$$

Thus, if we consider $F(K)$ as a function of r, then we have $F(K) > 0$ for all

$r > r_c^4$ and $F(K) < 0$ for all $r < r_c^4$.

Case B2. $1 \le r\theta \le 2 \Leftrightarrow \max\{1, r_c\theta\} \le r\theta \le 2$

For this case we have the relations

$$\frac{K}{r\theta} \le K \le \frac{2K}{r\theta}$$

and according to the formula $A_1 = 1 - W(0, A)$ and $A_2 = 1 - W(-1, A)$ which satisfies $1 < A_1 \le 2 \le A_2$. For this case, the relation between $r\theta$ and A_1 (or $r\theta$ and A_2) plays an important role. Note that A is a monotonically increasing function with respect to r, which indicates that A_1 is a monotonically decreasing function with respect to r, while A_2 is a monotonically increasing function with respect to r. Moreover, $A_1 = A_2 = 2$ at $r = r_c$ and $r_c\theta = \ln\left(\frac{\theta}{(1-\theta)p}\right) + 2$, which shows that $r_c\theta = 2$ when $p = \frac{\theta}{1-\theta}$. Furthermore, we have the following main results.

Lemma 4 $r_c\theta \le r\theta \le A_1 \le 2$ is equivalent to $r_c\theta \le r\theta \le r_c'\theta \le 2$, and $r_c\theta \le A_1 \le r\theta \le 2$ is equivalent to $r_c\theta \le r_c'\theta \le r\theta \le 2$. Moreover, $r_c = r_c'$ if and only if $p = \frac{\theta}{1-\theta}$, where $r_c' = \frac{1}{\theta}\left[\frac{\theta}{(1-\theta)p} + 1\right]$. $2 \le r_c\theta \le r\theta \le A_2$ is equivalent to $r\theta \ge r_c'\theta \ge r_c\theta \ge 2$, and $\max\{2, r_c\theta\} \le A_2 \le r\theta$ is equivalent to $\max\{2, r_c\theta\} \le r\theta \le r_c'\theta$

Proof If $r_c\theta \le r\theta \le A_1 \le 2$, then we first consider the equation

$$r\theta = A_1 = 1 - W(0, A), \text{ i.e., } W(0, A) = 1 - r\theta,$$

which indicates that

$$(1 - r\theta)e^{1-r\theta} = A = \frac{\theta}{(\theta - 1)pe^{r\theta-1}},$$

and solving the above equation with respect to r, yields

$$r=\frac{1}{\theta}\left[\frac{\theta}{(1-\theta)p}+1\right]\doteq r_c',$$

which indicates that $r\theta\le A_1$ is equivalent to $r\le r_c'$.

Denote

$$\begin{aligned}R_c &= r_c-r_c'=\tfrac{1}{\theta}\left[\ln\left(\tfrac{\theta}{(1-\theta)p}\right)+2\right]-\tfrac{1}{\theta}\left[\tfrac{\theta}{(1-\theta)p}+1\right]\\ &= \tfrac{1}{\theta}\left[\ln\left(\tfrac{\theta}{(1-\theta)p}\right)-\tfrac{\theta}{(1-\theta)p}+1\right].\end{aligned}$$

It is easy to show that $R_c\le 0$ (i.e., $r_c\le r_c'$) and equality holds true if and only if $\frac{\theta}{(1-\theta)p}=1$ (i.e., $p=\frac{\theta}{1-\theta}$). Similarly, we can show that the other results are true, and the proof is completed.

Lemma 5 The function $F(K/(r\theta))$ satisfies below properties: $F(K/(r\theta))<0$ for all $r\in(0,r_c^2)\cup(r_c^3,+\infty)$, $F(K/(r\theta))>0$ for all $r\in(r_c^2,r_c^3)$, and $F(K/(r\theta))=0$ at $r=r_c^2$ and r_c^3, where r_c^2 and r_c^3 are given in the following.

Proof If follows from the definition of the function $F(K/(r\theta))$ that we have $F(K/(r\theta))<0$ at $r=0$ and $\lim\limits_{r\to+\infty}F(K/(r\theta))=-1$. Moreover,

$$F(K/(r\theta))=pe^{r-1}e^{\frac{1}{A}}-1=0\Leftrightarrow pe^{r-1}=e^{-\frac{1}{A}}$$

which implies that

$$r-1+\ln(p)=-\frac{1}{A}=\frac{(1-\theta)pe^{r\theta-1}}{\theta}.$$

Rearranging the above equation, yields

$$-\left[r-1+\ln(p)\right]\theta e^{-[r-1+\ln(p)]\theta}=p(\theta-1)e^{-\ln(p)\theta+\theta-1}.$$

Therefore, according to the definition of the Lambert W function, if $-e^{-1}\le p(\theta-1)e^{-\ln(p)\theta+\theta-1}$, then we can solve the above equation with

respect to r, and the two roots r_c^2 and r_c^3 are given by

$$r_c^2 = 1 - \ln(p) - \frac{1}{\theta} W \left[0, p(\theta - 1)e^{-\ln(p)\theta + \theta - 1}\right]$$

and

$$r_c^3 = 1 - \ln(p) - \frac{1}{\theta} W \left[-1, p(\theta - 1)e^{-\ln(p)\theta + \theta - 1}\right].$$

In fact, the inequality $-e^{-1} \leq p(\theta - 1)e^{-\ln(p)\theta+\theta-1}$ is equivalent to $p(1 - \theta)e^{-\ln(p)\theta+\theta} \leq 1$. Denote the function $G(\theta) = p(1-\theta)e^{-\ln(p)\theta+\theta}$, then taking the derivative with respect to θ, one has

$$\frac{dG(\theta)}{d\theta} = pe^{-(\ln(p)-1)\theta} \left[\ln(p)\theta - \ln(p) - \theta\right].$$

Solving $\frac{dG(\theta)}{d\theta} = 0$ with respect to θ, yields the extreme point $\theta_m = \frac{\ln(p)}{\ln(p)-1}$, which satisfies $0 < \theta_m < 1$ for all $p \in (0, 1)$. This indicates that $G(\theta)$ is monotonically increasing for $\theta \in (0, \theta_m)$, and it is monotonically decreasing for $\theta \in (\theta_m, 1)$. Moreover, $G(\theta_m) = \frac{1}{1-\ln(p)} < 1$ for all $p \in (0, 1)$ with $G(0) = p$ and $G(1) = 0$, confirming that $-e^{-1} \leq p(\theta - 1)e^{-\ln(p)\theta+\theta-1}$ holds true naturally, and consequently, r_c^2 and r_c^3 are well defined. This completes the proof.

Note that there exists a unique $r_c^4 = \frac{-\ln(p)}{(1-p)(1-\theta)}$ such that $F(K) = 0$ at $r = r_c^4$. Moreover, $F(K) > 0$ for all $r > r_c^4$ and $F(K) < 0$ for all $r < r_c^4$.

Further, we consider the relation between $F(K)$ and $F(K/(r\theta))$.

Lemma 6 $F(K/(r\theta)) > F(K)$ when $r \in (r_c^5, r_c^6)$, $F(K/(r\theta)) < F(K)$ when $r \in (0, r_c^5) \cup (r_c^6, +\infty)$, and $F(K/(r\theta)) = F(K)$ at $r = r_c^5$ or $r = r_c^6$,

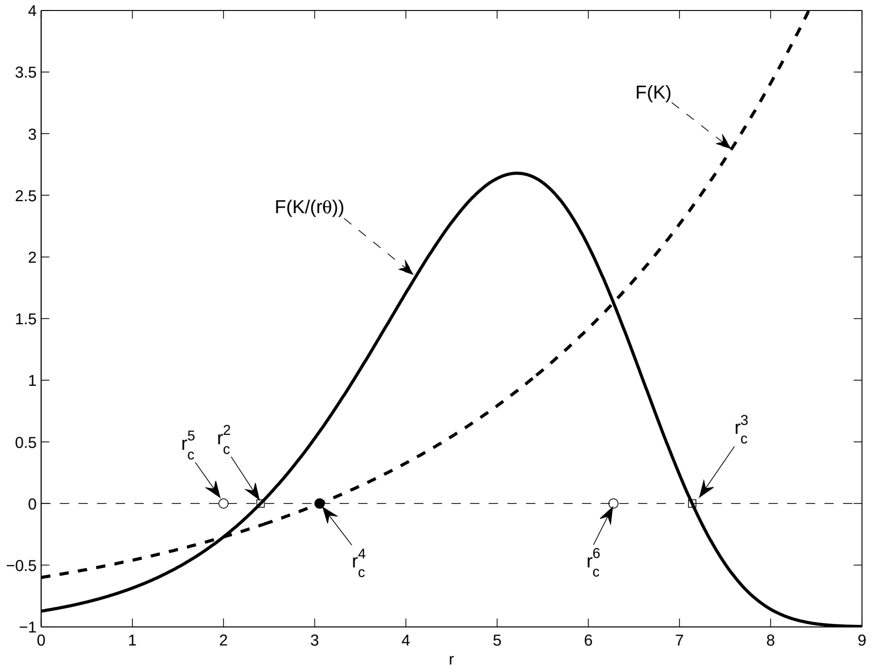

Figure S.4: Illustration of the relations between $F(K)$ and $F(K/(r\theta))$, here we just show one possible case.

where r_c^5 and r_c^6 are given in the following.

Proof It is easy to see that

$$\begin{aligned} F(K/(r\theta)) &= pe^{r(1-\theta)(1-p)}e^{r\theta+rp-r\theta p-1+\frac{1}{A}}-1 \\ &= [F(K)+1]\,e^{r\theta+rp-r\theta p-1+\frac{1}{A}}-1. \end{aligned}$$

We define the function

$$\begin{aligned} H(r) &= r\theta+rp-r\theta p-1+\tfrac{1}{A} \\ &= r(\theta+p-\theta p)-1-\tfrac{(1-\theta)pe^{r\theta-1}}{\theta}. \end{aligned}$$

Letting $F(K/(r\theta))=F(K)$, we have

$$[F(K)+1]\,e^{H(r)}-1=F(K),$$

that is,

$$F(K)(e^{H(r)}-1)=1-e^{H(r)}.$$

It follows from $F(K)>-1$ that $H(r)=0$.

Solving the equation $H(r)=0$ with respect to r, we obtain two roots

$$r_c^5=\frac{1}{\theta},\ r_c^6=\frac{1}{\theta(p+\theta-p\theta)}\left[(p\theta-p-\theta)W(-1,G_1)+\theta\right]>\frac{1}{\theta}$$

with

$$G_1=\frac{p(1-\theta)}{p\theta-p-\theta}e^{\frac{p(1-\theta)}{p\theta-p-\theta}},\ \text{and}\ -e^{-1}\le G_1<0.$$

If $r\in(r_c^5,r_c^6)$, then $H(r)>0$, thus $F(K/(r\theta))>F(K)$, if $r\in(0,r_c^5)\cup(r_c^6,+\infty)$, then $H(r)<0$, thus $F(K/(r\theta))<F(K)$. This completes the proof.

Therefore, based on the above discussions, we conclude that the relationships among those threshold values $r_c^2, r_c^3, r_c^4, r_c^5$ and r_c^6 are quite complex, and Fig.S.4 shows one of the possible cases.

Let

$$\begin{aligned} R_c^0 &= r_c - r_c^4 = \frac{1}{\theta}\left[\ln\left(\frac{\theta}{(1-\theta)p}\right) + 2\right] + \frac{\ln(p)}{(1-p)(1-\theta)} \\ &= \frac{\ln\left(\frac{\theta}{p(1-\theta)}\right)(1-p)(1-\theta)+\ln(p)\theta+2(1-p)(1-\theta)}{(1-p)(1-\theta)\theta}. \end{aligned}$$

and

$$\begin{aligned} R_c^1 &= r'_c - r_c^4 = \frac{1}{\theta}\left[\frac{\theta}{(1-\theta)p} + 1\right] + \frac{\ln(p)}{(1-p)(1-\theta)} \\ &= \frac{\ln(p)\theta p+(1-p)(\theta+p-\theta p)}{(1-p)(1-\theta)\theta p}. \end{aligned}$$

Define the function

$$G_1(p) = \ln(p)\theta p + (1-p)(\theta + p - \theta p).$$

It is easy to see that $\lim_{p\to 1^-} G_1(p) = 0$ and $\lim_{p\to 0^+} G_1(p) = \theta$. Moreover, $\frac{dG_1(p)}{dp} = \ln(p)\theta + (1-\theta)(1-2p)$, and solving $\frac{dG_1(p)}{dp} = 0$ with respect to p (the necessary condition for the existence of positive p is that $p < 1/2$), we obtain two roots

$$p_c^1 = \frac{\theta}{2(\theta-1)} W\left[0, \frac{2(\theta-1)}{\theta} e^{\frac{\theta-1}{\theta}}\right]$$

and

$$p_c^2 = \frac{\theta}{2(\theta-1)} W\left[-1, \frac{2(\theta-1)}{\theta} e^{\frac{\theta-1}{\theta}}\right],$$

provided that $\frac{2(\theta-1)}{\theta} e^{\frac{\theta-1}{\theta}} \geq -e^{-1}$. Note that there are two threshold values θ_c^1 and θ_c^2 such that $\frac{2(\theta-1)}{\theta} e^{\frac{\theta-1}{\theta}} < -e^{-1}$ for all $\theta \in (\theta_c^1, \theta_c^2)$. This indicates that p_c^1 and p_c^2 do not exist for all $\theta \in (\theta_c^1, \theta_c^2)$, and consequently, $G_1(p) > 0$ must hold true. Therefore, $R_c^1 > 0$ and $r'_c > r_c^4$ for all $\theta \in (\theta_c^1, \theta_c^2)$ and $p \in (0, 1/2)$.

Thus, based on the above discussions, the following two sub-cases should be further considered.

Case B2.1. $1 \leq r\theta \leq A_1$, i.e., $\max\{1, r_c\theta\} \leq r\theta \leq r'_c\theta \leq 2$

Based on the above inequalities, we easily have

$$\frac{K}{r\theta} \leq K \leq N_1 \leq \frac{2K}{r\theta} \leq N_2$$

and the results can be discussed as those in case B1. That is, we can obtain that if $F(N_1) > 0$, then equation $F(N) = 0$ has only one root N_3^* with $N_3^* > N_2 \geq K$; if $F(K) < 0$ and $F(N_2) < 0$, then equation $F(N) = 0$ has only one root N_1^* with $\frac{K}{r\theta} \leq N_1^* \leq K$; if $F(K) > 0$ and $F(N_2) < 0$, then equation $F(N) = 0$ has only one root N_1^* with $K < N_1^* < N_1$; if $F(K) < 0$ and $F(N_2) > 0$, then equation $F(N) = 0$ has three roots N_1^*, N_2^*, N_3^* with $N_1^* < K < N_1 < N_2^* < N_2 < N_3^*$; if $F(K) > 0$, $F(N_1) < 0$ and $F(N_2) > 0$, then equation $F(N) = 0$ has three roots N_1^*, N_2^*, N_3^* with $K < N_1^* < N_1 < N_2^* < N_2 < N_3^*$.

Case B2.2. $A_1 < r\theta \leq 2$, i.e., $r_c\theta \leq r_c'\theta < r\theta \leq 2$

In this case, we have the following relations:

$$\frac{K}{r\theta} \leq N_1 \leq K \leq \frac{2K}{r\theta} \leq N_2.$$

Thus, if $F(N_1) > 0$, then equation $F(N) = 0$ has only one root N_3^* with $N_3^* > N_2 > K$; if $F(0) > 0$ and $F(N_2) < 0$, then equation $F(N) = 0$ has only one root N_1^* with $N_1^* < N_1 < K$; if $F(N_1) < 0$ and $F(K) > 0$, then equation $F(N) = 0$ has three roots N_1^*, N_2^*, N_3^* with $N_1^* < N_1 < N_2^* < K < N_2 < N_3^*$; if $F(N_2) > 0$ and $F(K) < 0$, then equation $F(N) = 0$ has three roots N_1^*, N_2^*, N_3^* with $N_1^* < N_1 < K < N_2^* < N_2 < N_3^*$.

Case B3. $r\theta > 2$

If $r\theta > 2$, then we have

$$\frac{2K}{r\theta} < K.$$

By using methods similar to those used above, we consider the following two

sub-cases.

Case B3.1. $2 < r\theta \le A_2$, i.e., $r\theta \ge r_c'\theta \ge r_c\theta > 2$

Because $r\theta \le A_2$, therefore, $N_2 \ge K$. Thus, we have

$$\frac{K}{r\theta} \le N_1 \le \frac{2K}{r\theta} < K \le N_2.$$

Obviously, this case is the same as case B2.2 and can be similarly discussed.

Case B3.2. $r\theta > A_2$, i.e., $\max\{2, r_c\theta\} \le r\theta < r_c'\theta$

If $r\theta > A_2$ then $N_2 < K$. Thus, we have

$$\frac{K}{r\theta} \le N_1 \le \frac{2K}{r\theta} < N_2 < K.$$

Thus, if $F(N_1) > 0$ and $F(K) > 0$, then equation $F(N) = 0$ has only one root N_3^* with $N_3^* > K > N_2$; if $F(N_1) > 0$ and $F(K) < 0$, then equation $F(N) = 0$ has only one root N_3^* with $N_2 < N_3^* < K$; if $F(N_2) < 0$, then equation $F(N) = 0$ has only one root N_1^* with $N_1^* < N_1 < N_2 < K$;
if $F(N_1) < 0$, $F(N_2) > 0$ and $F(K) < 0$, then equation $F(N) = 0$ has three roots N_1^*, N_2^*, N_3^* with $N_1^* < N_1 < N_2^* < N_2 < N_3^* < K$; if $F(N_1) < 0$ and $F(K) > 0$, then equation $F(N) = 0$ has three roots N_1^*, N_2^*, N_3^* with $N_1^* < N_1 < N_2^* < N_2 < K < N_3^*$.

We list all possible cases for the existence of positive equilibria and their relations with respect to the carrying capacity in Tab.S.1.

3.1. The local stability of equilibrium N^*

Based on the above subsection, there are at most three positive roots for equation (S2.9), denoted by N_1^*, N_2^*, N_3^*, respectively.

Table S.1: Sufficient conditions for the existence of the positive equilibria $N_i^*, i = 1, 2, 3$

Cases		$F(N_1) > 0, F(N_2) > 0$		$F(N_1) < 0, F(N_2) > 0$		$F(N_1) < 0, F(N_2) < 0$	
		$F(K) < 0$	$F(K) > 0$	$F(K) < 0$	$F(K) > 0$	$F(K) < 0$	$F(K) > 0$
$r\theta < r_c\theta$		$N_1^* < K$	$N_1^* > K$				
$r_c\theta \leq r\theta < 1$		×	$N_3^* > K$	$N_1^* < K < N_2^* < N_3^*$	$K < N_1^* < N_2^* < N_3^*$	$N_1^* < K$	$N_1^* > K$
$r_c\theta_1 \leq r\theta \leq 2$	B2.1	×	$N_3^* > K$	$N_1^* < K < N_2^* < N_3^*$	$K < N_1^* < N_2^* < N_3^*$	$N_1^* < K$	$N_1^* > K$
	B2.2	×	$N_3^* > K$	$N_1^* < K < N_2^* < N_3^*$	$N_1^* < N_2^* < K < N_3^*$	$N_1^* < K$	×
$2 < r\theta$	B3.1	×	$N_3^* > K$	$N_1^* < K < N_2^* < N_3^*$	$N_1^* < N_2^* < K < N_3^*$	$N_1^* < K$	×
	B3.2	$N_3^* < K$	$N_3^* > K$	$N_1^* < N_2^* < N_3^* < K$	$N_1^* < N_2^* < K < N_3^*$	$N_1^* < K$	×

Note that for the first case $r\theta < r_c\theta$ we only need $F(K) < 0$ or $F(K) > 0$. $r_c\theta_1 = \max\{1, r_c\theta\}$, $r_c\theta_2 = \max\{2, r_c\theta\}$,$B2.1:\ r_c\theta_1 \leq r\theta \leq r_c'\theta \leq 2$, $B2.2:\ r_c\theta_1 \leq r_c'\theta < r\theta \leq 2$ $B3.1:\ 2 < r_c\theta \leq r_c'\theta \leq r\theta$, $B3.2:\ r_c\theta_2 \leq r\theta < r_c'\theta$.

Taking the derivative of $f(N)$ with respect to N, yields

$$f'(N) = pe^{g(N)} + pNe^{g(N)}g'(N),$$

and

$$f'(0) = pe^r.$$

Thus, if $pe^r > 1$, then N_0^* is unstable. This indicates that the pest population could be eradicated by spraying pesticides provided that the instant killing rate q is large enough (i.e., the survival rate p is small enough).

For the stability of positive equilibria N^*, we have

$$f'(N^*) = 1 + N^*g'(N^*).$$

It follows from the monotonicity of the function $F(N)$ that we have $F'(N) > 0$ for all $N \in (N_1, N_2)$, which indicates that $F'(N_2^*) > 0$ due to $N_2^* \in (N_1, N_2)$. It follows from (S2.10) that $g'(N_2^*) > 0$, and consequently we have $f'(N_2^*) > 1$. All these results confirm that N_2^* is unstable if it exists.

For the stabilities of N_1^* and N_3^*, we need $|f'(N^*)| < 1$, i.e., $-2 < N^* g'(N^*) < 0$. Note that if N_1^* (or N_3^*) or both are the positive equilibria of model (S1.13), then it follows from the proof of Theorem 1 that the signs of both $F'(N)$ and $g'(N)$ are equivalent, and then we conclude that $g'(N_1^*) < 0$ and $g'(N_3^*) < 0$. Therefore, in order to show the stabilities of both N_1^* and N_3^*, we only need to show whether the inequality $-2 < N^* g'(N^*)$ holds true or not. Thus, we first consider the following function $D(N)$, i.e.,

$$D(N) = -Ng'(N) - 2 = \frac{rN}{K}\left[\theta + \left(1 - \frac{r\theta}{K}N\right)h(N)\right] - 2. \qquad \text{(S3.12)}$$

Therefore, if $D(N^*) < 0$, then N^* is locally stable; if $D(N^*) > 0$, then N^* is unstable.

According to equation (S2.9) we can get $g(N) = -\ln p$ at equilibrium $N = N^*$. The function $D(N)$ plays an important role in analyzing the stability of the equilibrium, thus we do not distinguish the N and N^* when using the function $D(N)$ in the following. It follows from (S2.8) and (S2.9) that we have

$$r\left[1 - \frac{N}{K}(\theta + h(N))\right] = -\ln p.$$

Solving this equation for $h(N)$, we have

$$h(N) = \frac{K}{N}\left(1 + \frac{1}{r}\ln p\right) - \theta.$$

Substituting $h(N)$ into (S3.12), we can get

$$D(N) = \frac{r^2\theta^2}{K^2}N^2 - \frac{r\theta}{K}(r + \ln p)N + (r + \ln p) - 2.$$

Further, solving the equation $D(N) = 0$ with respect to N, yields two roots as follows

$$\bar{N}_1 = \frac{r + \ln p - \sqrt{(r + \ln p - 2)^2 + 4}}{\frac{2r\theta}{K}}$$

and

$$\bar{N}_2 = \frac{r + \ln p + \sqrt{(r + \ln p - 2)^2 + 4}}{\frac{2r\theta}{K}}.$$

Thus, based on the above discussion, we have $D(N) < 0$ for all $N \in (\bar{N}_1, \bar{N}_2)$, and $D(N) \geq 0$ for all $N \in (-\infty, \bar{N}_1] \cup [\bar{N}_2, +\infty)$.

It follows from $r + \ln(p) > 0$ (i.e., $pe^r > 1$) that $\bar{N}_2 > 0$, and it is easy to see that $r + \ln(p) - 2 > 0$ is equivalent to $\bar{N}_1 > 0$. Therefore, based on this equivalence, for the local stability of all possible positive equilibria we consider the following two cases.

Case S_1 $0 < r + \ln p \leq 2$

In this case, $\bar{N}_1 \leq 0$ and $D(N) < 0$ for all $N \in (0, \bar{N}_2)$. By a simple calculation, we have

$$D\left(\frac{2K}{r\theta}\right) = 2 - (r + \ln p) \geq 0$$

and $\bar{N}_2 = \frac{r+\ln p+\sqrt{(r+\ln p-2)^2+4}}{\frac{2r\theta}{K}} \leq \frac{4}{\frac{2r\theta}{K}} \leq 2K/(r\theta) \leq N_2$.

Let

$$\Omega^1_{r,p,\theta} = \left\{(r, p, \theta) \middle| r\theta + \ln \tfrac{1-\theta}{2\theta} > 1 - Z(r, p) - \ln p - \ln(-2Z(r, p)), \right.$$

$$\left. 0 < r + \ln p \leq 2, r \geq r_c \right\}$$

and

$$C_{\Omega^1_{r,p,\theta}} = \Big\{(r,p,\theta)\Big| r\theta + \ln\tfrac{1-\theta}{2\theta} \le 1 - Z(r,p) - \ln p - \ln(-2Z(r,p)),$$

$$0 < r + \ln p \le 2, r \ge r_c\Big\},$$

where

$$Z(r,p) \doteq -\frac{r + \ln(p) - 2 + \sqrt{(r + \ln p - 2)^2 + 4}}{2}.$$

Lemma 7 If $(r,p,\theta) \in \Omega^1_{r,p,\theta}$ then $N_1 < \bar{N}_2$, while if $(r,p,\theta) \in C_{\Omega^1_{r,p,\theta}}$ then $N_1 \ge \bar{N}_2$.

Proof We first consider the following equation $N_1 = \bar{N}_2$, i.e.,

$$\frac{K}{r\theta}\left[1 - W(0,A)\right] = \frac{r + \ln p + \sqrt{(r + \ln p - 2)^2 + 4}}{\frac{2r\theta}{K}}.$$

Rearranging the above equation gives

$$1 - W(0,A) = \frac{r + \ln p + \sqrt{(r + \ln p - 2)^2 + 4}}{2},$$

which indicates that

$$W(0,A) = -\frac{r + \ln(p) - 2 + \sqrt{(r + \ln p - 2)^2 + 4}}{2} = Z(r,p). \qquad \text{(S3.13)}$$

Moreover, for all $0 < r + \ln(p) \le 2$, we have

$$-1 \le Z(r,p) = -\frac{r + \ln(p) - 2 + \sqrt{(r + \ln p - 2)^2 + 4}}{2} < 1 - \sqrt{2},$$

and this shows that equation (S3.13) is well defined according to the domain of the Lambert W function. Thus, we can obtain the following relation:

$$Z(r,p)e^{Z(r,p)} = A = \frac{\theta}{(\theta - 1)pe^{r\theta - 1}},\ r \ge r_c,\ -1 \le Z(r,p) < 1 - \sqrt{2}, \quad \text{(S3.14)}$$

from which the upper branch of $W(0,A)$ of (S3.14) with respect to $Z(r,p)$ is well defined and reveals the relationship among three parameters r, p, θ such that $N_1 = \bar{N}_2$.

Therefore,

$$
\begin{aligned}
N_1 < \bar{N}_2 \quad &\Leftrightarrow \quad W(0,A) > Z(r,p) \Leftrightarrow A > Z(r,p)e^{Z(r,p)} \\
&\Leftrightarrow \quad \tfrac{\theta}{(\theta-1)pr^{r\theta-1}} > \tfrac{2-(r+\ln p)-\sqrt{(r+\ln p-2)^2+4}}{2}\exp\left(\tfrac{2-(r+\ln p)-\sqrt{(r+\ln p-2)^2+4}}{2}\right) \\
&\Leftrightarrow \quad \tfrac{2-(r+\ln p)-\sqrt{(r+\ln p-2)^2+4}}{2}\tfrac{(\theta-1)p}{\theta} > \exp\left(1-r\theta-\tfrac{2-(r+\ln p)-\sqrt{(r+\ln p-2)^2+4}}{2}\right) \\
&\Leftrightarrow \quad \begin{array}{c} \ln\left(\tfrac{1-\theta}{2\theta}\right) + \ln p + \ln\left(-2+(r+\ln p)+\sqrt{(r+\ln p-2)^2+4}\right) \\ > \ 1-r\theta-\tfrac{2-(r+\ln p)-\sqrt{(r+\ln p-2)^2+4}}{2} \end{array} \\
&\Leftrightarrow \quad (r,p,\theta) \in \Omega^1_{r,p,\theta}
\end{aligned}
$$

and

$$N_1 \geq \bar{N}_2 \Leftrightarrow (r,p,\theta) \in C_{\Omega^1_{r,p,\theta}}.$$

This completes the proof.

It follows from $\bar{N}_2 \leq 2K/(r\theta) \leq N_2 < N_3^*$ that $D(N_3^*) > 0$, which indicates that N_3^* is unstable if it exists, and further according to $N_1^* \leq N_1$ we have the following main results.

Theorem 2 For Case S_1 if $(r,p,\theta) \in \Omega^1_{r,p,\theta}$, then $N_1 < \bar{N}_2$, thus, $N_1^* < \bar{N}_2$, and consequently, $D(N_1^*) < 0$. This indicates that N_1^* is locally stable;

similarly, if $(r,p,\theta) \in C_{\Omega^1_{r,p,\theta}}$ and $N_1^* < \bar{N}_2$ then N_1^* is also locally stable. However, if $(r,p,\theta) \in C_{\Omega^1_{r,p,\theta}}$ and $\bar{N}_2 \leq N_1^*$, then N_1^* is unstable if it exists. However, N_3^* is unstable if it exists for Case S_1.

Case S_2 $r + \ln p - 2 > 0$

For this case, we have $Z(r,p) < -1$ and $\bar{N}_1 > 0$. Thus, by a simple calculation, we have

$$D(0) = (r + \ln p) - 2 > 0$$

and

$$D\left(\frac{2K}{r\theta}\right) = 2 - (r + \ln p) < 0.$$

It follows from

$$D\left(\frac{K}{r\theta}\right) = -1, \text{ and } \frac{K}{r\theta} < N_1 < \frac{2K}{r\theta} < N_2$$

that we have $D(N_1) < 0$. Thus, we can get $\bar{N}_1 < \frac{K}{r\theta} < N_1 < \frac{2K}{r\theta} < N_2$. It follows from $N_1^* < N_1 < N_2 < N_3^*$ that if $\bar{N}_1 < N_1^* < N_1$, then $D(N_1^*) < 0$, which indicates that N_1^* is locally stable. If $N_1^* \leq \bar{N}_1$, then $D(N_1^*) \geq 0$, this indicates that $N_1^* g'(N_1^*) < -2$. That is $f'(N_1^*) < -1$, which indicates that N_1^* is unstable if it exists.

In order to determine the stability of N_3^*, we need the relations between N_2 and $\bar{N}_2$. Let

$$\Omega^2_{r,p,\theta} = \left\{(r,p,\theta)\middle| r\theta + \ln\tfrac{1-\theta}{2\theta} < 1 - Z(r,p) - \ln p - \ln(-2Z(r,p)),\right.$$
$$\left. r + \ln p > 2, r > r_c\right\}$$

and

$$C_{\Omega^2_{r,p,\theta}} = \left\{(r,p,\theta)\middle| r\theta + \ln\tfrac{1-\theta}{2\theta} \geq 1 - Z(r,p) - \ln p - \ln(-2Z(r,p)),\right.$$

$$\left. r + \ln p > 2, r > r_c\right\},$$

where

$$Z(r,p) \doteq -\frac{r + \ln(p) - 2 + \sqrt{(r + \ln p - 2)^2 + 4}}{2}.$$

By employing the same methods as those in Lemma 7, we have the following results.

Lemma 8 If $(r,p,\theta) \in \Omega^2_{r,p,\theta}$ then $N_2 < \bar{N}_2$, while if $(r,p,\theta) \in C_{\Omega^2_{r,p,\theta}}$ then $N_2 \geq \bar{N}_2$.

Theorem 3 *If $(r,p,\theta) \in C_{\Omega^1_{r,p,\theta}}$ (i.e., $N_2 \geq \bar{N}_2$), then $N_3^* > \bar{N}_2$, $D(N_3^*) > 0$ and $f'(N_3^*) < -1$, which shows that N_3^* is unstable if it exists.*
If $(r,p,\theta) \in \Omega^2_{r,p,\theta}$ (i.e., $N_2 < \bar{N}_2$) and $N_3^* > \bar{N}_2$, then $D(N_3^*) > 0$ and $f'(N_3^*) < -1$, which indicates that N_3^* is unstable if it exists.
If $(r,p,\theta) \in \Omega^2_{r,p,\theta}$ (i.e., $N_2 < \bar{N}_2$) and $N_2 < N_3^* < \bar{N}_2$, then $D(N_3^*) < 0$, which indicates that N_3^* is locally stable if it exists.

We list all possible cases for the stability of positive equilibria and their parameter spaces in Tab.S.2.

Table S.2: The local stabilities of N_1^* and N_3^*.

Cases		$0 < r + \ln p < 2$	$r + \ln p \geq 2$
Stable	N_1^*	$(r, p, \theta) \in \Omega^1_{r,p,\theta}$ or $(r, p, \theta) \in C_{\Omega^1_{r,p,\theta}}$, $N_1^* < \bar{N}_2$	$\bar{N}_1 < N_1^* < N_1$
	N_3^*	It does not exist	$(r, p, \theta) \in \Omega^2_{r,p,\theta}$, $N_3^* < \bar{N}_2$
Unstable	N_1^*	$(r, p, \theta) \in C_{\Omega^1_{r,p,\theta}}$, $N_1^* \geq \bar{N}_2$	$N_1^* \leq \bar{N}_1$
	N_3^*	Unstable if it exists	$(r, p, \theta) \in C_{\Omega^2_{r,p,\theta}}$ or $(r, p, \theta) \in \Omega^2_{r,p,\theta}$, $N_3^* > \bar{N}_2$

4. Hormesis and paradox with respect to parameters p and θ

Differentiating Eq. (S2.9) with respect to p and letting it equal to 0, we have

$$e^{g(N^*)} + pe^{g(N^*)}\left(\frac{\partial g}{\partial N^*}\frac{\partial N^*}{\partial p} + \frac{\partial g}{\partial p}\right) = 0. \tag{S4.15}$$

Substituting Eq. (S2.9) into (S4.15), we can get

$$\frac{1}{p} + \frac{\partial g}{\partial N^*}\frac{\partial N^*}{\partial p} + \frac{\partial g}{\partial p} = 0. \tag{S4.16}$$

It follows from (S2.7) and (S2.8) that we have

$$\frac{\partial g}{\partial p} = -\frac{rN^*}{Kp}h(N^*) \tag{S4.17}$$

and

$$\frac{\partial g}{\partial N^*} = -\frac{r}{K}\left[\theta + \left(1 - \frac{r\theta N^*}{K}\right)h(N^*)\right]. \tag{S4.18}$$

All these results confirm that

$$\frac{\partial N^*}{\partial p} = \frac{1 - \frac{rN^*}{K}h(N^*)}{\frac{pr}{K}\left[\theta + \left(1 - \frac{r\theta N^*}{K}\right)h(N^*)\right]}. \tag{S4.19}$$

According to Eq.(S2.9), we have

$$-\ln(p) = g(N^*) = r - \frac{r\theta N^*}{K} - \frac{rN^*}{K}h(N^*),$$

i.e., we have

$$h(N^*) = \frac{r + \ln p - \frac{r\theta N^*}{K}}{\frac{rN^*}{K}}. \tag{S4.20}$$

Therefore,

$$\begin{aligned}\frac{\partial N^*}{\partial p} &= \frac{1}{p}\frac{N^*\left[\frac{r\theta N^*}{K} - (r + \ln p - 1)\right]}{\left(\frac{r\theta}{K}\right)^2 (N^*)^2 - \frac{r\theta}{K}(r + \ln p)N^* + r + \ln p} \\ &= \frac{1}{p}\frac{N^*\left[N^* - \bar{N}_0\right]}{\bar{D}(N^*)}\end{aligned}$$

with

$$\bar{N}_0 = \frac{r + \ln p - 1}{\frac{r\theta}{K}}$$

and

$$\bar{D}(N^*) = \left(\frac{r\theta}{K}\right)^2 (N^*)^2 - \frac{r\theta}{K}(r + \ln p)N^* + r + \ln p.$$

Denote

$$\Delta_{Np} = \left(\frac{r\theta}{K}\right)^2 (r + \ln(p))(r + \ln(p) - 4)$$

and it follows from $r+\ln(p) > 0$ that $\Delta_{Np} \geq 0$ provided that $r+\ln(p)-4 \geq 0$.

For convenience, we denote $x = r + \ln(p) = -a$. Thus, if $x - 4 \geq 0$, then for the equation $\bar{D}(N^*) = 0$ two roots exist, i.e.,

$$\bar{N}_{3,4} = \frac{x \mp \sqrt{x(x-4)}}{\frac{2r\theta}{K}}, \tag{S4.21}$$

which implies that $\bar{D}(N^*) > 0$ for all $N^* \in (0, \bar{N}_3) \cup (\bar{N}_4, +\infty)$ and $\bar{D}(N^*) < 0$ for all $N^* \in (\bar{N}_3, \bar{N}_4)$. However, if $x - 4 < 0$, then $\bar{D}(N^*) > 0$ for all $N^* > 0$.

Lemma 9 $\bar{N}_1 < \bar{N}_0 < \bar{N}_2$. If $x - 4 \geq 0$, then $\bar{N}_1 < \bar{N}_3 \leq \bar{N}_4 < \bar{N}_0 < \bar{N}_2$.

Proof. According to the expressions of $\bar{N}_1$ and $\bar{N}_0$, we have

$$\begin{aligned}\bar{N}_1 - \bar{N}_0 &= \frac{x-\sqrt{(x-2)^2+4}}{\frac{2r\theta}{K}} - \frac{x-1}{\frac{r\theta}{K}} \\ &= \frac{-\sqrt{(x-2)^2+4}-(x-2)}{\frac{2r\theta}{K}},\end{aligned}$$

thus, if $x - 2 \geq 0$, then $\bar{N}_1 - \bar{N}_0 < 0$, that is $\bar{N}_1 < \bar{N}_0$.

If $x - 2 < 0$, then we have

$$\begin{aligned}\bar{N}_1 = \frac{x-\sqrt{(x-2)^2+4}}{\frac{2r\theta}{K}} &< \frac{x-(2-r-\ln p)}{\frac{2r\theta}{K}} \\ &= -\frac{x-1}{\frac{r\theta}{K}} = \bar{N}_0.\end{aligned}$$

According to the expressions of $\bar{N}_2$ and $\bar{N}_0$, we have

$$\begin{aligned}\bar{N}_2 - \bar{N}_0 &= \frac{x+\sqrt{(x-2)^2+4}}{\frac{2r\theta}{K}} - \frac{x-1}{\frac{r\theta}{K}} \\ &= \frac{\sqrt{(x-2)^2+4}-(x-2)}{\frac{2r\theta}{K}} > 0.\end{aligned}$$

Therefore, $\bar{N}_1 < \bar{N}_0 < \bar{N}_2$.

For $x - 4 \geq 0$, it follows from

$$\sqrt{(x-2)^2+4} > \sqrt{(x)(x-4)}$$

that

$$\bar{N}_1 = \frac{x-\sqrt{(x-2)^2+4}}{\frac{2r\theta}{K}}$$

$$< \frac{x-\sqrt{(x)(x-4)}}{\frac{2r\theta}{K}} = \bar{N}_3.$$

Due to $\bar{N}_2 > \bar{N}_0$ and $\bar{N}_0 > \bar{N}_4 \geq \bar{N}_3$, we can conclude that $\bar{N}_2 > \bar{N}_0 > \bar{N}_4 \geq \bar{N}_3 > \bar{N}_1$. This completes the proof.

However, if $x - 4 \geq 0$, then we have $\bar{N}_0 > \bar{N}_4 \geq \bar{N}_3$. Thus, if $N^* > \bar{N}_0$, then we have $\frac{\partial N^*}{\partial p} > 0$; if $\bar{N}_0 > N^* > \bar{N}_4$, then $\frac{\partial N^*}{\partial p} < 0$; if $\bar{N}_0 > \bar{N}_4 > N^* > \bar{N}_3$, then $\frac{\partial N^*}{\partial p} > 0$; if $\bar{N}_0 > \bar{N}_4 > \bar{N}_3 > N^*$, then $\frac{\partial N^*}{\partial p} < 0$.

Theorem 4 If $x - 4 < 0$, then $\frac{\partial N^*}{\partial p} > 0$, provided that $N^* > \bar{N}_0$. Moreover, if $\bar{N}_0 < 0$ (i.e., $0 < x < 1$), then $\frac{\partial N^*}{\partial p} > 0$ holds true once N^* exists; if $x - 4 < 0$ then $\frac{\partial N^*}{\partial p} \leq 0$, provided that $N^* \leq \bar{N}_0$.

If $x - 4 \geq 0$, then $\frac{\partial N^*}{\partial p} > 0$ provided that $\bar{N}_3 < N^* < \bar{N}_4$ or $N^* > \bar{N}_0$; $\frac{\partial N^*}{\partial p} < 0$ provided that $\bar{N}_4 < N^* < \bar{N}_0$ or $0 < N^* < \bar{N}_3$; $\frac{\partial N^*}{\partial p} = 0$ provided that $N^* = \bar{N}_0$ or $N^* = 0$.

The effects of parameter q or p on the positive equilibria, hormetic and paradoxical effects are listed in the Tab.S.3.

Now we turn our attention to the effects of θ on N^*.

Differentiating Eq. (S2.9) with respect to θ, we have

$$pe^{g(N^*)}\left(\frac{\partial g}{\partial N^*}\frac{\partial N^*}{\partial \theta} + \frac{\partial g}{\partial \theta}\right) = 0. \qquad \text{(S4.22)}$$

Substituting Eq. (S2.9) into (S4.22), we can get

$$\frac{\partial g}{\partial N^*}\frac{\partial N^*}{\partial \theta} + \frac{\partial g}{\partial \theta} = 0,$$

Table S.3: Hormetic and paradoxical effects of θ on the equilibrium N^*, and the sufficient conditions of the effects of p on N^* for all possible cases.

Cases	Paradoxical effects of survival rate p on N^*					
$0 < r + \ln(p) \leq 1$	$(0, \bar{N}_2)$	$[\bar{N}_2, +\infty)$				
	S & $\frac{\partial N^*}{\partial p} > 0$	US & $\frac{\partial N^*}{\partial p} > 0$				
$1 < r + \ln(p) \leq 2$	$(0, \bar{N}_0]$	$(\bar{N}_0, \bar{N}_2)$	$[\bar{N}_2, +\infty)$			
	S & $\frac{\partial N^*}{\partial p} \leq 0$	S& $\frac{\partial N^*}{\partial p} > 0$	US & $\frac{\partial N^*}{\partial p} > 0$			
$2 < r + \ln(p) < 4$	$(0, \bar{N}_1]$	$(\bar{N}_1, \bar{N}_0]$	$(\bar{N}_0, \bar{N}_2)$	$[\bar{N}_2, +\infty)$		
	US & $\frac{\partial N^*}{\partial p} < 0$	S & $\frac{\partial N^*}{\partial p} \leq 0$	S & $\frac{\partial N^*}{\partial p} > 0$	US & $\frac{\partial N^*}{\partial p} > 0$		
$r + \ln(p) \geq 4$	$(0, \bar{N}_1]$	$(\bar{N}_1, \bar{N}_3)$	$(\bar{N}_3, \bar{N}_4)$	$(\bar{N}_4, \bar{N}_0)$	$(\bar{N}_0, \bar{N}_2)$	$[\bar{N}_2, +\infty)$
	US & $\frac{\partial N^*}{\partial p} < 0$	S & $\frac{\partial N^*}{\partial p} < 0$	S & $\frac{\partial N^*}{\partial p} > 0$	S & $\frac{\partial N^*}{\partial p} < 0$	S & $\frac{\partial N^*}{\partial p} > 0$	US & $\frac{\partial N^*}{\partial p} > 0$

that is,

$$\frac{\partial N^*}{\partial \theta} = -\frac{\frac{\partial g}{\partial \theta}}{\frac{\partial g}{\partial N^*}} = \frac{N^* \frac{\partial g}{\partial \theta}}{\bar{D}(N^*)}. \tag{S4.23}$$

By a simple calculation, we have

$$
\begin{aligned}
\frac{\partial g}{\partial \theta} &= -\tfrac{rN^*}{K}\left(1+\tfrac{\partial h}{\partial \theta}\right) \\
&= \tfrac{rN^*}{K}\left[1+h(N^*)\left(r(1-\tfrac{N^*}{K})-\tfrac{1}{1-\theta}\right)\right] \\
&= -\left(\tfrac{r}{K}\right)^2\theta(N^*)^2+\tfrac{r}{K}\left[\theta(r-\tfrac{1}{1-\theta})+r+\ln p-1\right]N^*-(r+\ln p)(r-\tfrac{1}{1-\theta}) \\
&\doteq g_\theta(N^*).
\end{aligned}
\tag{S4.24}
$$

Letting $g_\theta(N^*)=0$, we have

$$-\left(\frac{r}{K}\right)^2\theta(N^*)^2+\frac{r}{K}\left[\theta\left(r-\frac{1}{1-\theta}\right)+r+\ln p-1\right]N^*-(r+\ln p)\left(r-\frac{1}{1-\theta}\right)=0.$$

Denote

$$
\begin{aligned}
\Delta_{g_\theta} &= (\tfrac{r}{K})^2\left[\theta(r-\tfrac{1}{1-\theta})+r+\ln p-1\right]^2-4\left(\tfrac{r}{K}\right)^2\theta(r+\ln p)(r-\tfrac{1}{1-\theta}) \\
&= (\tfrac{r}{K})^2\left[\left(\theta(r-\tfrac{1}{1-\theta})-(r+\ln p-1)\right)^2-4\theta(r-\tfrac{1}{1-\theta})\right].
\end{aligned}
$$

Now we discuss the sign of Δ_{g_θ}.

Denote

$$Z=Z(r,\theta)\doteq\theta\left(r-\frac{1}{1-\theta}\right).$$

Hence

$$\begin{aligned}\Delta_{g_\theta} &= (\tfrac{r}{K})^2\,[Z^2 - 2(r+\ln p+1)Z + (r+\ln p-1)^2]\\ &= (\tfrac{r}{K})^2(Z-Z_1)(Z-Z_2),\end{aligned}$$

where

$$Z_1 = (\sqrt{r+\ln p}-1)^2, \quad Z_2 = (\sqrt{r+\ln p}+1)^2.$$

Therefore, if $Z < Z_1$ or $Z > Z_2$, then $\Delta_{g_\theta} > 0$; if $Z_1 < Z < Z_2$, then $\Delta_{g_\theta} < 0$.

According to the above discussion, we can conclude that

(i_θ) if $Z_1 < Z < Z_2$, then $g_\theta(N^*) < 0$;

(ii_θ) if $Z < Z_1$ or $Z > Z_2$, there are two roots $\bar{N}_5$ and $\bar{N}_6$ of equation $g_\theta(N^*) = 0$. And if $N^* < \bar{N}_5$ or $N^* > \bar{N}_6$, then $g_\theta(N^*) < 0$; if $\bar{N}_5 < N^* < \bar{N}_6$ then $g_\theta(N^*) > 0$, where

$$\bar{N}_5 = \frac{x+Z-1-\sqrt{(x+Z-1)^2-4Zx}}{2(\frac{r\theta}{K})},$$

$$\bar{N}_6 = \frac{x+Z-1+\sqrt{(x+Z-1)^2-4Zx}}{2(\frac{r\theta}{K})}.$$

Note that, if $Z < 0$, then $\bar{N}_5 < 0$.

Substituting (S4.18) and (S4.24) into (S4.23), we have

$$\begin{aligned}\frac{\partial N^*}{\partial \theta} &= \frac{g_\theta(N^*)}{\frac{r}{K}\left[\theta + \left(1-\frac{r\theta N^*}{K}\right)h(N^*)\right]} = \frac{N^* g_\theta(N^*)}{\bar{D}(N^*)}\\ &= -\frac{N^*(N^*-\bar{N}_5)(N^*-\bar{N}_6)}{(\frac{r\theta}{K})^2(N^*-\bar{N}_3)(N^*-\bar{N}_4)} \text{ (if } N_i \text{ exist).}\end{aligned}$$

Therefore, if $x < 4$, and $Z_1 < Z < Z_2$, then $\frac{\partial N^*}{\partial \theta} < 0$; if $x < 4$, and $Z < Z_1$ or $Z < Z_2$, then $\frac{\partial N^*}{\partial \theta} > 0$ when $\bar{N}_5 \leq N^* \leq \bar{N}_6$, $\frac{\partial N^*}{\partial \theta} \leq 0$ when $\bar{N}_5 > N^*$ or $N^* > \bar{N}_6$.

Now we discuss the relations among $\bar{N}_i (i = 3, 4, 5, 6)$ under the case of $x > 4$.

Lemma 10 If $x > 4$, then
$\bar{N}_5 < \bar{N}_3 \leq \bar{N}_4 < \bar{N}_6$, when $Z < 1$;
$\bar{N}_3 < \bar{N}_5 \leq \bar{N}_6 < \bar{N}_4$, when $1 < Z < Z_1$;
$\bar{N}_3 < \bar{N}_4 < \bar{N}_5 < \bar{N}_6$, when $Z > Z_2$;
$\bar{N}_3 = \bar{N}_5$ and $\bar{N}_6 = \bar{N}_4$, when $Z = 1$.

Proof. Let

$$w_{35}(Z) \doteq \frac{2r\theta}{K}(\bar{N}_3 - \bar{N}_5) = \sqrt{(x + Z - 1)^2 - 4Zx} - \sqrt{x^2 - 4x} - (Z - 1). \tag{S4.25}$$

Therefore, if $Z \leq 1 - \sqrt{x^2 - 4x}$, then $w_{35} > 0$, that is, $\bar{N}_3 > \bar{N}_5$.

Now we consider the case of $Z > 1 - \sqrt{x^2 - 4x}$. Under this case, we have

$$\begin{aligned} \left(\frac{Z-1+\sqrt{x^2-4x}}{\sqrt{(x+Z-1)^2-4Zx}}\right)^2 &= \frac{x^2-4x+(Z-1)^2+2(Z-1)\sqrt{x^2-4x}}{(x+Z-1)^2-4Zx} \\ &= \frac{(x+Z-1)^2-2Zx-2x+2(Z-1)\sqrt{x^2-4x}}{(x+Z-1)^2-4Zx}. \end{aligned}$$

The ranges for the existence of the $\bar{N}_i$ $(i = 3, 4, 5, 6)$ are $Z \in (-\infty, Z_1] \cup [Z_2, +\infty)$ with $x \geq 4$ and $Z_1 = (1 - \sqrt{x})^2 \geq 1$.

If $Z \leq 1$, then

$$\left(\frac{Z - 1 + \sqrt{x^2 - 4x}}{\sqrt{(x + Z - 1)^2 - 4Zx}}\right)^2 \leq \frac{(x + Z - 1)^2 - 4Zx}{(x + Z - 1)^2 - 4Zx} = 1,$$

therefore, $\bar{N}_3 \geq \bar{N}_5$ when $1 - \sqrt{x^2 - 4x} < Z \leq 1$.

Thus, $\bar{N}_3 \geq \bar{N}_5$ when $Z \leq 1$.

If $1 < Z < Z_1$ or $Z > Z_2$, then

$$\left(\frac{Z - 1 + \sqrt{x^2 - 4x}}{\sqrt{(x + Z - 1)^2 - 4Zx}} \right)^2 > \frac{(x + Z - 1)^2 - 4Zx}{(x + Z - 1)^2 - 4Zx} = 1.$$

Thus, $\bar{N}_3 < \bar{N}_5$, when $1 < Z < Z_1$ or $Z > Z_2$. And $\bar{N}_3 = \bar{N}_5$, when $Z = 1$.

Letting

$$w_6(Z) = x + Z - 1 + \sqrt{(x + Z - 1)^2 - 4xZ},$$

$$Z \in (-\infty, Z_1] \cup [Z_2, +\infty), \; x \geq 4,$$

we have

$$\bar{N}_6 = \frac{w_6(Z)}{\frac{2r\theta}{K}}, \; \bar{N}_4 = \frac{w_6(1)}{\frac{2r\theta}{K}},$$

and

$$w'(Z) = 1 + \frac{Z - 1 - x}{\sqrt{(x + Z - 1)^2 - 4xZ}} = 1 + \frac{Z - 1 - x}{\sqrt{(x - Z - 1)^2 - 4x}}.$$

If $Z > Z_2$, then $Z - 1 - x > 0$, thus $w'(Z) > 0$ and $w(Z)$ is increasing; and if $Z < Z_1$, then $Z - 1 - x < 0$, thus $w'(Z) < 0$ and $w(Z)$ decreasing.

Because $Z_2 > 1$, therefore, $\bar{N}_6 > \bar{N}_4$, when $Z \geq Z_2$. Due to $Z_1 < 1$, thus, $\bar{N}_6 > \bar{N}_4$, when $Z < Z_1$, $\bar{N}_6 < \bar{N}_4$, when $1 < Z \leq Z_1$, and $\bar{N}_6 = \bar{N}_4$, when $Z = 1$.

Let

$$w_{45}(Z) \doteq \frac{2r\theta}{K}(\bar{N}_4 - \bar{N}_5) = \sqrt{(x - Z + 1)^2 - 4x} + \sqrt{x^2 - 4x} - (Z - 1), \quad \text{(S4.26)}$$

with $Z \geq Z_2, x \geq 4$.

Because

$$Z - 1 - \sqrt{x^2 - 4x} > Z - 1 - x \geq 2\sqrt{x} > 0,$$

and

$$\begin{aligned} \left(Z - 1 - \sqrt{x^2 - 4x}\right)^2 &= (Z-1)^2 + x^2 - 4x - 2(Z-1)\sqrt{x^2 - 4x} \\ &> (Z-1)^2 + x^2 - 4x - 2(Z-1)x \\ &= \left(\sqrt{(x - Z + 1)^2 - 4x}\right)^2, \end{aligned}$$

thus $Z - 1 - \sqrt{x^2 - 4x} < \sqrt{(x - Z + 1)^2 - 4x}$, that is $w_{45}(Z) < 0$, which indicates that $\bar{N}_4 < \bar{N}_5$.

Therefore,

$\bar{N}_5 < \bar{N}_3 \leq \bar{N}_4 < \bar{N}_6$, when $Z < 1$;

$\bar{N}_3 < \bar{N}_5 \leq \bar{N}_6 < \bar{N}_4$, when $1 < Z < Z_1$;

$\bar{N}_3 < \bar{N}_4 < \bar{N}_5 < \bar{N}_6$, when $Z > Z_2$;

$\bar{N}_3 = \bar{N}_5$ and $\bar{N}_6 = \bar{N}_4$, when $Z = 1$.

This completes the proof. In summary, we have the following main results.

Theorem 5 **Case** (I_θ) $Z < 1, x > 4$

(1) $\frac{\partial N^*}{\partial \theta} < 0$ when $0 < N^* < \bar{N}_5$ or $\bar{N}_3 < N^* < \bar{N}_4$ or $N^* > \bar{N}_6$;

(2) $\frac{\partial N^*}{\partial \theta} > 0$ when $\bar{N}_5 < N^* < \bar{N}_3$ or $\bar{N}_4 < N^* < \bar{N}_6$.

Case (II_θ) $1 < Z < Z_1, x > 4$

(1) $\frac{\partial N^*}{\partial \theta} < 0$ when $0 < N^* < \bar{N}_3$ or $\bar{N}_5 < N^* < \bar{N}_6$ or $N^* > \bar{N}_4$;

(2) $\frac{\partial N^*}{\partial \theta} > 0$ when $\bar{N}_3 < N^* < \bar{N}_5$ or $\bar{N}_6 < N^* < \bar{N}_4$.

Case (III_θ) $Z > Z_2, x > 4$

(1) $\frac{\partial N^*}{\partial \theta} < 0$ when $0 < N^* < \bar{N}_3$ or $\bar{N}_4 < N^* < \bar{N}_5$ or $N^* > \bar{N}_6$;

(2) $\frac{\partial N^*}{\partial \theta} > 0$ when $\bar{N}_3 < N^* < \bar{N}_4$ or $\bar{N}_5 < N^* < \bar{N}_6$.

Case (IV_θ) $Z = 1, x > 4$

In this case, $\frac{\partial N^*}{\partial \theta} \leq 0$ for all $N^* > 0$.

Case $(V_\theta) x > 4$, and $N^* = \bar{N}_5$ or $N^* = \bar{N}_6$

In this case, $\frac{\partial N^*}{\partial \theta} = 0$.

The effects of parameter θ on the positive equilibria, hormetic and paradoxical effects are listed in Tab.S.3.

5. Multiple control actions within each generation

Without loss of generality, we assume that the chemical control tactics have been applied m times within each generation $[n, n+1]$, i.e., there exist $\theta_i (i = 1, 2, \cdots, m)$ with $n \leq n + \theta_1 \leq n + \theta_2 \leq \cdots \leq n + \theta_m \leq n + 1$, such that the control measures have been applied at $n + \theta_i$ with a proportion q_i of the pest being killed. Thus, the survival rate after each chemical control is $p_i = 1 - q_i$. For convenience, we denote $\theta_0 = 0$, $\theta_{m+1} = 1$ and $p_0 = 1$.

Therefore, by employing the same methods proposed before we can extend the Beverton-Holt model with multiple controls within each generation as follows

$$N_{n+1} = \frac{\left(\prod_{j=1}^{m} p_j\right) RKN_n}{\sum_{j=1}^{m+1} (R^{\theta_j} - R^{\theta_{j-1}}) \left(\prod_{k=1}^{j} p_{k-1}\right) N_n + K}. \qquad \text{(S5.27)}$$

In particular, if $m = 1$ then we have $\sum_{j=1}^{m+1} \left(R^{\theta_j} - R^{\theta_{j-1}}\right) \left(\prod_{k=1}^{j} p_{k-1}\right) N_n =$

$(R^{\theta_1} - 1)N_n + (R - R^{\theta_1})p_1 N_n$.

The Ricker model with multiple controls within each generation can be generalized as follows

$$N_{n+1} = \left(\prod_{i=1}^{m} p_i\right) N_n A_{m+1}. \tag{S5.28}$$

$$A_{m+1} = \exp\left(r - \frac{r}{K}N_n\left(\theta_1 + \sum_{i=1}^{m}(\theta_{i+1} - \theta_i)A_i\left(\prod_{j=1}^{i} p_j\right)\right)\right)$$

and

$$A_i = \exp\left(r\theta_i - \frac{r}{K}N_n\left(\theta_1 + \sum_{j=1}^{i-1}(\theta_{j+1} - \theta_j)A_j\left(\prod_{l=1}^{j} p_l\right)\right)\right)$$

In particular, we have

$$A_1 = \exp\left(r\left(1 - \frac{N_n}{K}\right)\theta_1\right), \ A_2 = \exp\left(r\theta_2 - \frac{r}{K}N_n\left(\theta_1 + (\theta_2 - \theta_1)p_1 A_1\right)\right)$$

$$A_3 = \exp\left(r\theta_3 - \frac{r}{K}N_n\left(\theta_1 + (\theta_2 - \theta_1)p_1 A_1 + (\theta_3 - \theta_2)p_1 p_2 A_2\right)\right)$$

and

$$A_4 = \exp\left(r\theta_4 - \frac{r}{K}N_n\left(\theta_1 + (\theta_2 - \theta_1)p_1 A_1 + (\theta_3 - \theta_2)p_1 p_2 A_2 + (\theta_4 - \theta_3)p_1 p_2 p_3 A_3\right)\right).$$

Therefore, if $m = 1$ then we have $\theta_2 = 1$ and

$$N_{n+1} = N_{n+1} = \left(\prod_{i=1}^{m} p_i\right) N_n A_2 = p_1 N_n \exp\left(r\theta_2 - \frac{r}{K}N_n\left(\theta_1 + (\theta_2 - \theta_1)p_1 A_1\right)\right).$$

6. Some important definitions related to the Lambert *W* function

Definition The Lambert W function is defined to be a multivalued inverse of the function $z \mapsto ze^z$ satisfying

$$LambertW(z)\exp(LambertW(z)) = z.$$

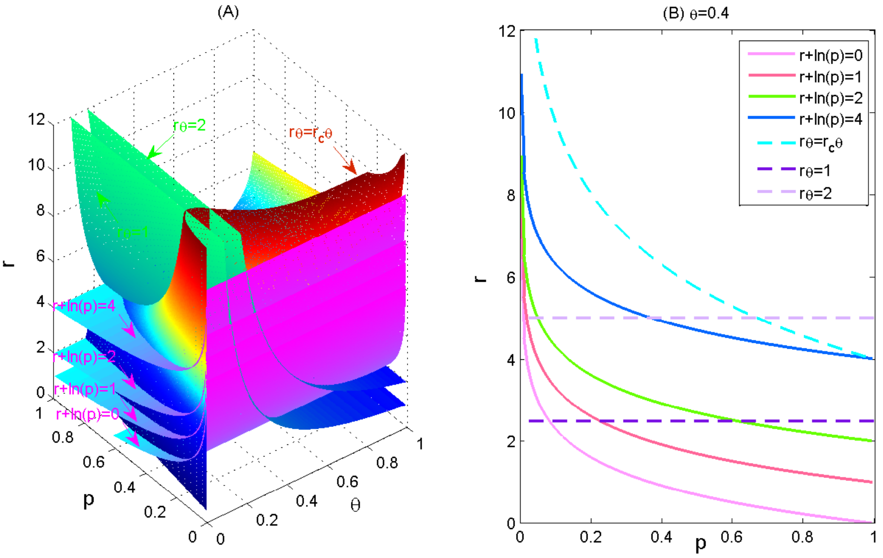

Figure S.5: Three-parameter and two-parameter bifurcations show the complex parameter spaces for the cases listed in Tables SI.1, SI.2, SI.3 and SI.4.

For simplicity, we denote it by W. Note that if $z > -1$ then the function $z\exp(z)$ has the positive derivative $(z+1)\exp(z)$. Define the inverse function of $z\exp(z)$ restricted on the interval $[-1, \infty)$ to be $W(0, z) \doteq W(z)$. Similarly, we define the inverse function of $z\exp(z)$ restricted on the interval $(-\infty, -1]$ to be $W(-1, z)$. The $W(z)$ and $W(-1, z)$ are the two real branches of the Lambert W function. The branch $W(z)$ is defined on the interval $[-e^{-1}, +\infty)$, and it is a monotonically increasing function with respect to z, while the branch $W(-1, z)$ is defined on the interval $[-e^{-1}, 0)$, and it is a monotonically decreasing function with respect to z. Note that both branches are defined in the common interval $[-e^{-1}, 0)$ with $W(z) > W(-1, z)$ for

$z \in (-e^{-1}, 0)$, $W(-e^{-1}) = W(-1, -e^{-1}) = -1$ and $W(e) = 1$, as shown in Fig.S.6.

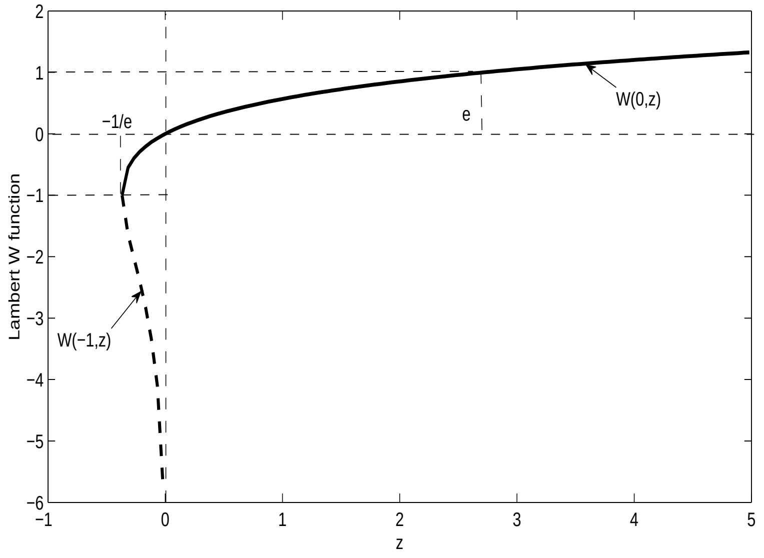

Figure S.6: The two real branches of the Lambert W function, $W(0, z)$ and $W(-1, z)$, and their domains.

[1] May, R. M. Biological populations obeying difference equations: stable points, stable cycles, and chaos. *J. Theor. Biol.* **51**(2),511-524(1975).

[2] May R.M. Simple mathematical models with very complicated dynamics. *Nature* **261**,459-467(1976).

[3] May, R.M., & Oster, G.F. Bifurcations and dynamic complexity in simple ecological models. *Amer. Natur.* 573-599(1976).

[4] Panetta, J.C. A mathematical model of periodically pulsed chemothera-

py: tumor recurrence and metastasis in a competitive environment. *Bull. Math. Biol.* **58** , 425-447(1996).

[5] Tang, S.Y., & Cheke, R.A. State-dependent impulsive models of integrated pest management (IPM) strategies and their dynamic consequences. *J. Math. Biol.* **50**, 257-292(2005).

[6] Beverton, R.J. & Holt S.J. The theory of fishing. In: M. Graham, ed., Sea Fisheries; their investigation in the United Kigdom. Edward Arnold, London 372-441(1956).

[7] Seno, H. A paradox in discrete single species population dynamics with harvesting/thinning, *Math. Biosci.* **214**,63-69(2008).

[8] Matsuoka, T. & Seno, H. Ecological balance in the native population dynamics may cause the paradox of pest control with harvesting. *J. Theor. Biol.* **252**,87-97(2008).

[9] Cid, B., Hilker, F.M. & Liz, E. Harvest timing and its population dynamic consequences in a discrete single-species model. *Math. Biosci.* **248**,78-87(2014).

Table S.4: Hormetic and paradoxical effects of time factor θ on the equilibrium N^*, and the sufficient conditions for the effects of θ on the sign of $\frac{\partial N^*}{\partial \theta}$.

<table>
<tr><td rowspan="3">$r + \ln(p) > 4$</td><td colspan="5">$Z \doteq Z(r,\theta) < 1$</td><td>$Z = 1$</td><td colspan="5">$1 < Z < Z_1$</td><td>$Z_1 \leq Z \leq Z_2$</td><td colspan="5">$Z > Z_2$</td></tr>
<tr><td>$0 < N^* < \bar{N}_5$</td><td>$\bar{N}_3 < N^* < \bar{N}_4$</td><td>$N^* > \bar{N}_6$</td><td>$\bar{N}_5 < N^* < \bar{N}_3$</td><td>$\bar{N}_4 < N^* < \bar{N}_6$</td><td rowspan="2">$\frac{\partial N^*}{\partial \theta} < 0$</td><td>$0 < N^* < \bar{N}_3$</td><td>$\bar{N}_5 < N^* < \bar{N}_6$</td><td>$N^* > \bar{N}_4$</td><td>$\bar{N}_3 < N^* < \bar{N}_5$</td><td>$\bar{N}_6 < N^* < \bar{N}_4$</td><td rowspan="2">$\frac{\partial N^*}{\partial \theta} \leq 0$</td><td>$0 < N^* < \bar{N}_3$</td><td>$N^* > \bar{N}_6$</td><td>$\bar{N}_4 < N^* < \bar{N}_5$</td><td>$\bar{N}_3 < N^* < \bar{N}_4$</td><td>$\bar{N}_5 < N^* < \bar{N}_6$</td></tr>
<tr><td colspan="3">$\frac{\partial N^*}{\partial \theta} < 0$</td><td colspan="2">$\frac{\partial N^*}{\partial \theta} > 0$</td><td colspan="3">$\frac{\partial N^*}{\partial \theta} < 0$</td><td colspan="2">$\frac{\partial N^*}{\partial \theta} > 0$</td><td colspan="3">$\frac{\partial N^*}{\partial \theta} < 0$</td><td colspan="2">$\frac{\partial N^*}{\partial \theta} > 0$</td></tr>
<tr><td rowspan="3">$r + \ln(p) < 4$</td><td colspan="11">$Z \doteq Z(r,\theta) < Z_1$</td><td rowspan="3">$\frac{\partial N^*}{\partial \theta} \leq 0$</td><td colspan="5">$Z > Z_2$</td></tr>
<tr><td colspan="5">$\bar{N}_5 < N^* < \bar{N}_6$</td><td colspan="6">$N^* > \bar{N}_6$ or $N^* < \bar{N}_5$</td><td colspan="3">$\bar{N}_5 < N^* < \bar{N}_6$</td><td>$N^* > \bar{N}_6$</td><td>$N^* < \bar{N}_5$</td></tr>
<tr><td colspan="5">$\frac{\partial N^*}{\partial \theta} > 0$</td><td colspan="6">$\frac{\partial N^*}{\partial \theta} \leq 0$</td><td colspan="3">$\frac{\partial N^*}{\partial \theta} > 0$</td><td colspan="2">$\frac{\partial N^*}{\partial \theta} \leq 0$</td></tr>
</table>

Note that if $r + \ln(p) > 4$ and $N^* = \bar{N}_5$ (or $N^* = \bar{N}_6$), then $\frac{\partial N^*}{\partial \theta} = 0$.
